# Green Synthesis of Aromatic Nitrogen-Containing Heterocycles by Catalytic and Non-Traditional Activation Methods

**DOI:** 10.3390/molecules28104153

**Published:** 2023-05-17

**Authors:** R. Bernadett Vlocskó, Guoshu Xie, Béla Török

**Affiliations:** Department of Chemistry, University of Massachusetts Boston, 100 Morrissey Blvd., Boston, MA 02125, USA

**Keywords:** sustainable synthesis, aromatic N-heterocycles, solid acids, nanoparticles, microwaves, ultrasounds, visible light activation, high hydrostatic pressure, electrochemistry, biomass

## Abstract

Recent advances in the environmentally benign synthesis of aromatic N-heterocycles are reviewed, focusing primarily on the application of catalytic methods and non-traditional activation. This account features two main parts: the preparation of single ring N-heterocycles, and their condensed analogs. Both groups include compounds with one, two and more N-atoms. Due to the large number of protocols, this account focuses on providing representative examples to feature the available methods.

## 1. Introduction

Heterocycles are a broad variety of compounds including aromatic and non-aromatic compounds with various heteroatoms, most commonly N, O and S. Many of them are natural compounds and a large majority are biologically active. They are used in large quantities in the pharmaceutical, agrochemical, dyestuff or polymer industries. Accordingly, the preparation and functionalization of heterocycles have attracted overwhelming interest [1,2,3]. The high-volume industrial production of heterocycles, however, brings about a serious environmental issue; the traditional processes yield large amounts of toxic waste and often represent hazardous conditions. Both must be avoided if at all possible, leading recent synthesis development efforts into the realm of green chemistry. These methods are effective, and at the same time they also comply with contemporary guidelines for safety and environmental sustainability [4,5]. Due to the extremely high activity in this field, the advances toward the sustainable production of heterocycles have been reported in thousands of papers. In this work we will survey the green synthesis of nitrogen-containing heterocycles focusing on representative example protocols that apply either solid catalysts [6,7,8] and/or non-traditional activation methods [9] that significantly decrease the amount of waste as well as the energy need of these processes. We mostly focused on the last five years from 2017–2023. However, occasionally representative examples were cited from earlier periods. Given that a large majority of the derivatives of aromatic N-heterocycles are biologically active, the question of chirality arose naturally. Due to the aromaticity of these heterocycles, there were no examples where the ring itself had any chiral center. Hence we could not include such examples. There are many papers that report chiral derivatives of N-heterocycles. However, all of them were prepared by the functionalization of the core heterocycles, and functionalization was beyond the scope of this work.

## 2. Five-Membered Rings

### 2.1. One-Nitrogen-Containing Heterocycles: Pyrroles

**Scheme 1:**
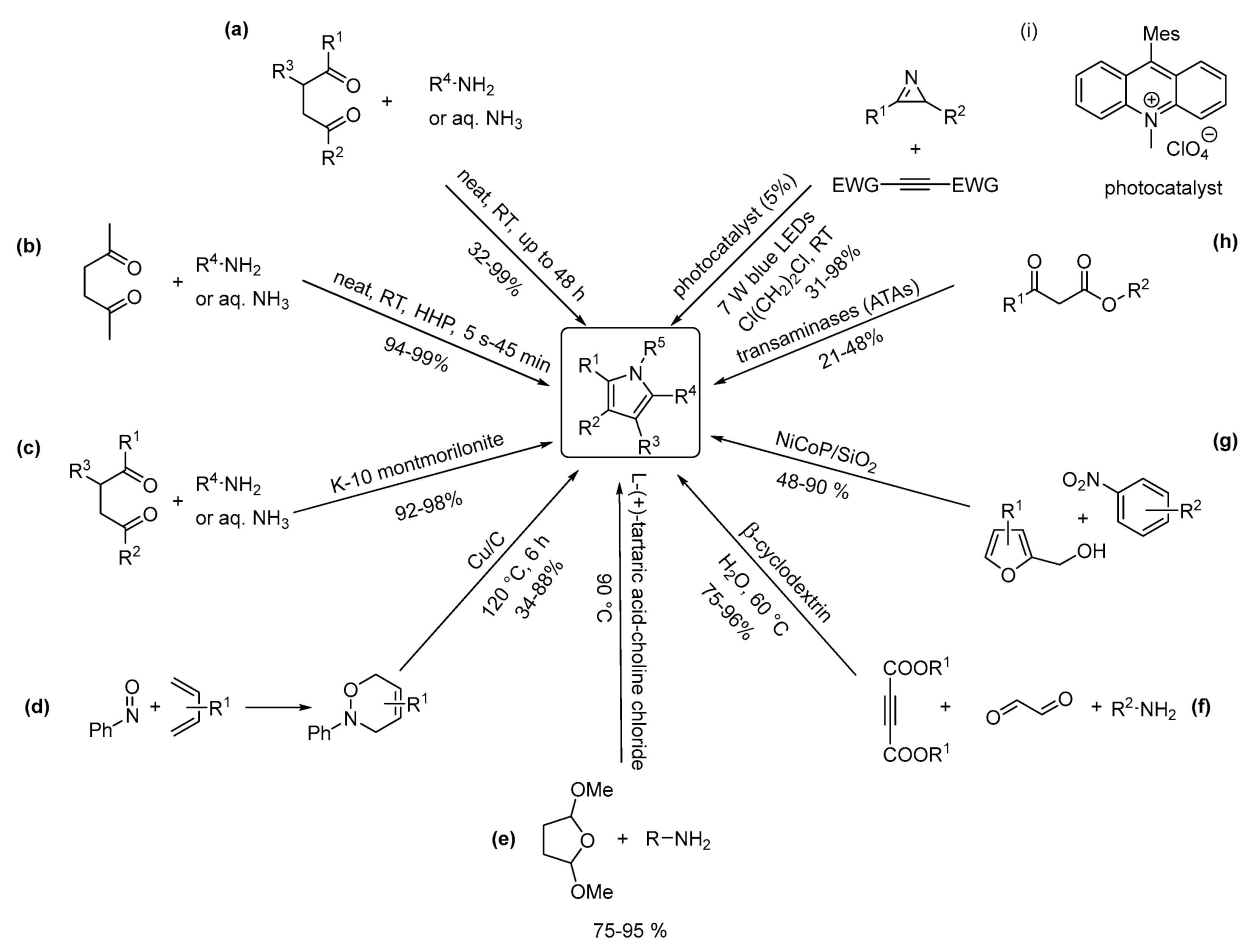
Representative green protocols for the synthesis of pyrroles.

The Paal–Knorr reaction [10,11], is one of the most well-known preparation methods to obtain pyrroles. Although effective in producing the desired pyrrole derivatives, the use of mineral acid catalysis renders it obsolete. There have been several attempts to make this reaction more environmentally benign [12]. A summary of representative green protocols is depicted in Scheme 1.

The greenest examples describe acid and solvent-free protocols that produce the pyrroles in high yields using 1,4-diketones and amines or aq. NH_4_OH. One process even occurs at room temperature, although the required reaction times are quite long (up to 24 h) (Scheme 1a) [13]. A recent protocol, applying similar starting materials at ambient temperature and using high hydrostatic pressure as an activation method, significantly reduced the reaction time to 5 s–45 min, while maintaining quantitative yields (Scheme 1b) [14]. A similar acid-free method utilized water as a solvent, which is abundant and non-toxic, though its recycling has a high energy demand [15]. Another green solvent (MeOH) was applied in the catalyst-free coupling of nitroolefins with enaminoesters, producing multi-substituted pyrroles in good yields [16]. Other reports include solid-acid catalysis by K-10 montmorillonite (Scheme 1c) [17] and even extend the scope to monosubstituted N-alkylpyrroles and N-sulfonyl-pyrroles using 2,5-dimethoxy- tetrahydrofuran to replace the diketones [18,19]. Most of the solid-acid-catalyzed protocols use microwave irradiation as activation [20,21]. Other heterogeneous catalytic methods used carbon-supported copper (Cu/C) to promote a hetero Diels–Alder reaction of nitroso dienophiles and 1,3-dienes in good yields (Scheme 1d) [22]. 2,5-Dimethoxy- tetrahydrofuran can also undergo cyclization with amines in deep eutectic solvents (DES) (Scheme 1e) [23]. The DES also acted as an organocatalyst and provided stable yields in five consecutive reactions. Similarly to DES, ionic liquids [24] can also catalyze the reaction. Anilines, acetylenedicarboxylic acid esters and glyoxal underwent a multicomponent cyclization catalyzed by β-cyclodextrin to afford pyrroles (Scheme 1f) [25]. β-Cyclodextrin, which is well-known for forming inclusion complexes [26] provides an appropriate nonpolar space in its cavity while the highly polar external wall keeps the catalyst–substrate complex stable in the aqueous medium. Essentially, β-cyclodextrin acted as a phase transfer catalyst and remained reusable in four subsequent reactions. The biomass-product furan alcohols reacted efficiently with nitrobenzenes over the surface of a bifunctional catalyst, metal phosphides (Scheme 1g) [27]. The catalyst possessed dual active sites namely solid acidic and metallic sites for ring opening, and the abstraction of H from the starting material and the in situ hydrogenation of the nitrobenzenes to anilines. The mixed NiCoP appeared to be the best catalyst providing the N-arylpyrroles in moderate to good yields. Another green example is a biocatalytic process using transaminases (ATAs) (Scheme 1h) [28]. In this protocol, α-diketones were aminated in a classical Knorr pyrrole synthesis. The pH of this biocatalytic system must be closely monitored to avoid the dimerization of the α-amino carbonyl intermediate. An emerging activation method, visible light LEDs, was applied in the synthesis of pyrroles. In general, visible-light-LED-assisted processes are widely applied in the synthesis of many heterocycles and are the target of frequent reviews [29,30,31,32]. In a photoredox reaction 2-azirines were combined with internal alkynes to afford pyrroles [33]. The energy from blue light LEDs utilized the intrinsic strain of 2-azirines to initiate a [3+2] cycloaddition with the alkyne in the presence of a 9-mesityl-10-methylacridinium perchlorate photocatalyst (Scheme 1i). The protocol provides a broad scope of pyrroles with moderate to excellent yields.

### 2.2. Two-Nitrogen-Containing Heterocycles: Pyrazoles and Imidazoles

**Scheme 2:**
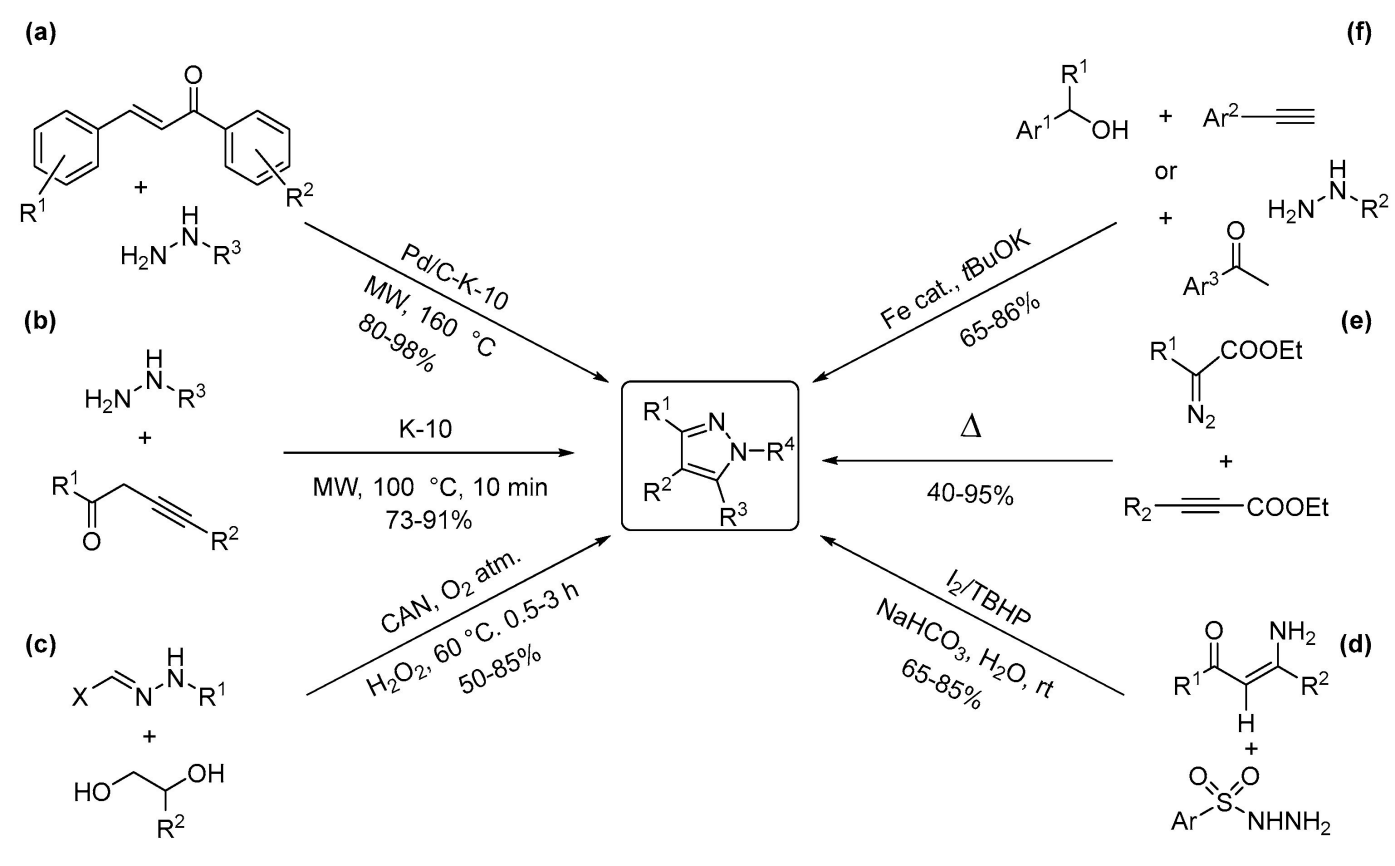
Representative green protocols for the synthesis of pyrazoles.

Pyrazoles are two N-containing five-membered aromatic heterocycles, possessing widespread biological effects and are crucial building blocks. Thus, their preparation is at the forefront of synthesis research, focusing on contemporary green and sustainable approaches that include catalytic, multicomponent and solvent-free protocols [34,35,36,37] as described in several reviews [38,39,40]. The classic synthesis of pyrazoles relies on the domino reaction of hydrazines with 1,3-bifunctional substrates often suffering from low regioselectivity. Representative recent advances are illustrated in Scheme 2.

The above-mentioned classic route was modified to a solid-acid-catalyzed process. The cyclization and aromatization of chalcones and arylhydrazones were catalyzed by Pd/C and K-10 montmorillonite and afforded the products in excellent yields [41]. The solid acid K-10 initiated the cyclization and then Pd oxidized the intermediate to pyrazoles (Scheme 2a). The same setup worked well with alk-3-yn-1-ones as well (Scheme 2b) [42]. In another catalytic method, cerium ammonium nitrate (CAN) was applied as a catalyst in the regioselective tandem oxidation and intermolecular ring cyclization of vicinal diols with hydrazones to pyrazoles (Scheme 2c) [43]. The products were isolated in moderate to excellent yields under mild conditions in an aqueous medium. Another hydrazine-based annulation protocol was also carried out in aqueous medium in a metal-free system (Scheme 2d) [44]. Molecular iodine was applied as a catalyst in the presence of *tert*-butylhydroperoxide and NaHCO_3_. The reaction occurred through domino C–H sulfonylation and annulation steps with good functional group tolerance affording the pyrazoles in good to excellent yields. The thermal cycloaddition of diazo compounds with alkynes occurred in a catalyst- and reagent-free system with moderate to excellent yields (Scheme 2e). The α-diazocarbonyl compounds readily formed the pyrazole derivatives in high yields in a simple solvent-free thermal reaction eliminating work-up or purification steps [45]. An efficient iron-catalyzed multicomponent synthesis of trisubstituted pyrazoles was developed using biomass-derived alcohols in an Fe(II)-catalyzed process (Scheme 2f) [46]. This protocol possessed a broad scope that was achieved by dehydrogenative coupling of alcohols, aryl hydrazines and secondary alcohols or using alkynes as an alternative to the *sec*-alcohols. This protocol eliminated the need for pre-functionalization, toxic noble metal catalysts, harmful oxidants or other additives. A recent protocol applied a silver-catalyzed [3 + 2] cycloaddition of aryl diazonium salts and allenes, providing the target pyrazoles in moderate to excellent yields, however, in high selectivity [47]. A similar approach, using aryl diazonium salts with arylcyclopropanols produced the target pyrazoles by a photocatalytic cycloaddition [48].

**Scheme 3:**
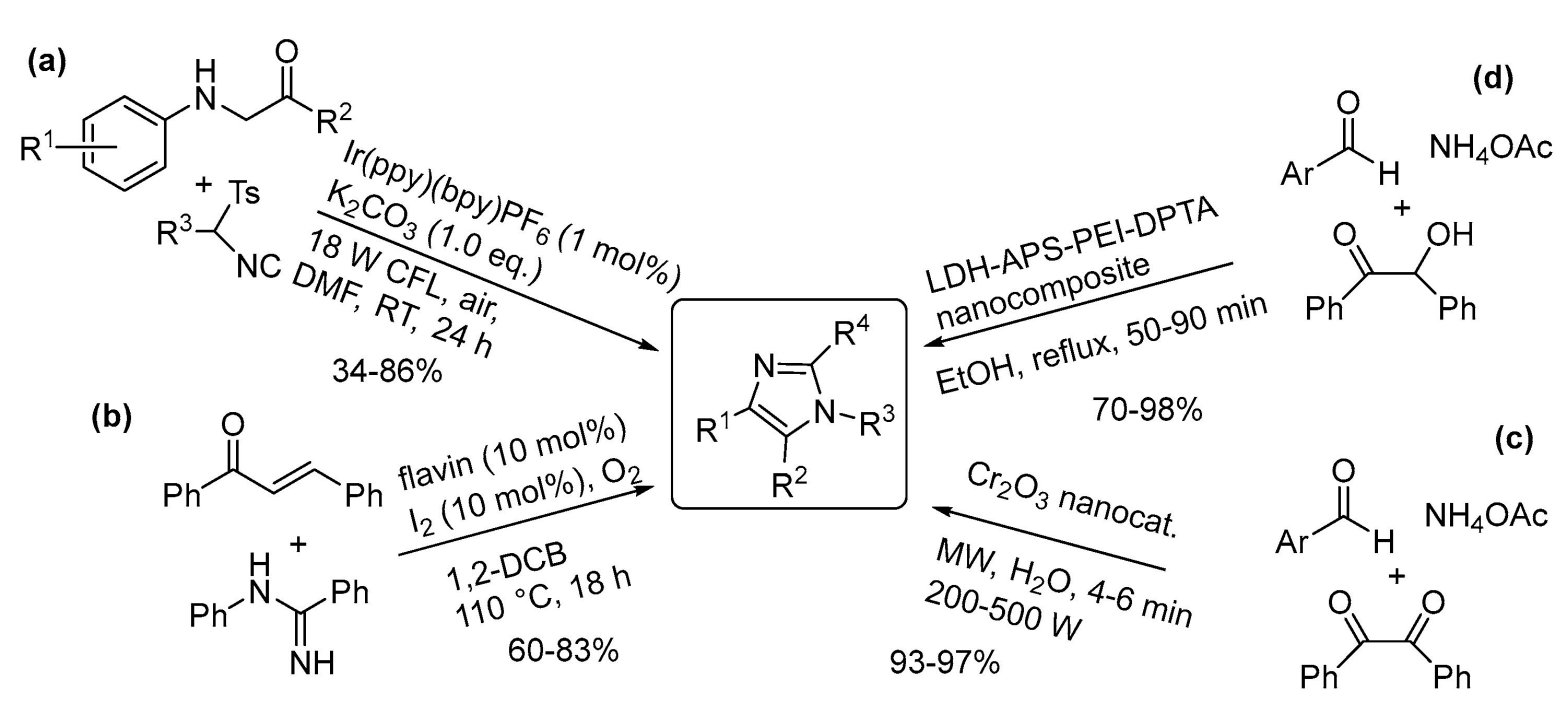
Representative green protocols for the synthesis of imidazoles.

The green synthesis of imidazoles also attracted extensive attention. A few representative examples are depicted in Scheme 3.

The visible-light-assisted photochemical processes have also been applied for the synthesis of imidazoles. The [3+2] cycloaddition-photooxidative aromatization sequence of glycine derivatives and isocyanides provided moderate to good yields for the synthesis of trisubstituted imidazoles (Scheme 3a) [49]. The reaction conditions were mild (rt etc.) and the protocol could be scaled up to a gram scale. A metal-free organocatalytic synthesis of imidazoles was carried out from amidines and chalcones. A natural product, flavin (with iodine), was used as a catalyst for the cross-dehydrogenative coupling (Scheme 3b) [50]. The reaction produced tetrasubstituted imidazoles in good yields (60−87%) in an atom economic design, consuming one equivalent of O_2_ and producing water as the only byproduct in an otherwise waste-free protocol. A heterogeneous catalytic synthesis of imidazoles was designed by using the Cr_2_O_3_ nanoparticle-catalyzed reaction of aromatic aldehydes with ammonium acetate and benzil under microwave-assisted conditions in water as solvent (Scheme 3c) [51]. The protocol offers operational simplicity, short reaction times and excellent yields. Another heterogeneous catalytic method applied nanoarchitectonics of LDH/polymer (layered double hydroxide) composite, and LDH/polymer nanocomposites (LDH–APS–PEI–DTPA). The polymer portion of the catalysts was prepared from diethylenetriaminepentaacetic acid (DTPA), polyethylenimine and used LDH to form a nanocomposite with high thermal stability. The product nanocomposite is an active and recyclable (five times) heterogeneous catalyst for the synthesis of imidazoles (Scheme 3d) [52].

### 2.3. Three-Nitrogen-Containing Heterocycles: Triazoles

**Figure 1:**
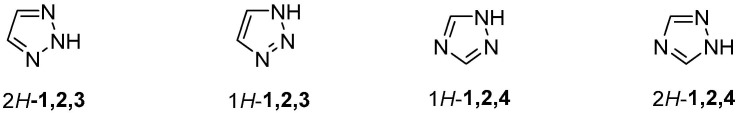
Various isomeric forms of triazole.

Triazoles are five-membered heterocycles with three nitrogen atoms and two double bonds. Depending on the position of the nitrogens and the double bonds, four triazole isomers can be distinguished (Figure 1). All triazole isomers are planar and aromatic.

**Scheme 4:**
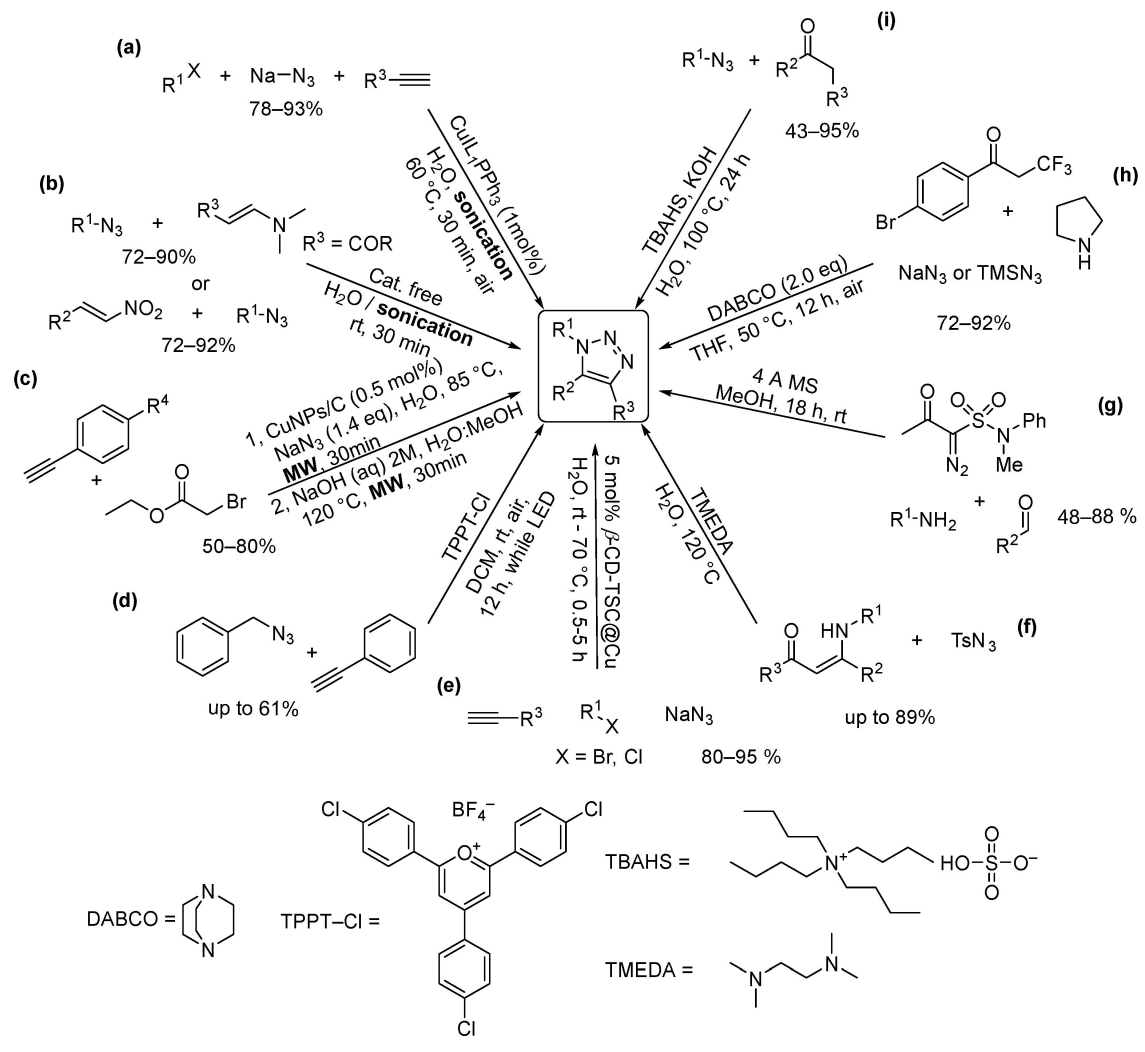
Representative green strategies for synthesis of triazoles.

Many of the 1,2,3- and 1,2,4-triazole derivatives show extensive pharmacological activities such as antifungal (fluconazole), herbicide (propiconazole), anticancer (carboxyamidotriazole) and antibacterial (cefatrizine), just to mention a few [53,54,55,56,57]. Not only are they good pharmacophores, but they have a wide range of applications in polymer and paint production [58,59,60]. The physiological and industrial significance of nitrogen-containing heterocycles, including triazoles, can be explained by their unique electronic and coordination properties, not to mention their H-bonding affinity due to which they can form many weak non-bonded or secondary interactions. One of the most widely applied synthesis methods to generate triazole and other heterocyclic derivatives is ‘click chemistry’. These are easy-to-perform, high-yielding stereospecific chemical transformations with wide substrate scope creating only innocuous by-products that can be removed without chromatography. These reactions in most cases are conducted in benign solvents, preferably in water and result in only one product. As we can see, the principles of click and green chemistry overlap in many ways [61]. Several known reactions meet these criteria such as addition reactions to C-C double or triple bonds, hydrazone formation and cycloaddition reactions. The predominant synthesis protocol for triazole synthesis has been, and still is, the use of organic azides and terminal alkynes as building blocks in a cycloaddition reaction, such as the classic 1,3-dipolar cycloaddition [62]. Unfortunately, this reaction requires elevated temperature and at the end of the reaction a mixture of the 1,4-substituted and the 1,5-substituted regioisomers is generated. The reaction was improved by adding a Cu(I) salt to the system, which accelerated it to 10^7^–10^8^ times the original rate and was selective in favor of the 1,4- isomer [63]. Later Fokin and Jia introduced the use of Ru-based catalysts in cycloaddition reactions which selectively leads to the 1,5-disubstituted 1,2,3-triazole isomer [64]. Recently Hong et al. also described a highly selective 1,5-disubstituted triazole synthesis using Cp_2_Ni/Xantphos catalytic system [65]. Herein we present the latest trends and innovations of sustainable synthesis of relevant triazoles. Representative examples are shown in Scheme 4.

Most of the listed reactions (Scheme 4a,b,e,f,i) are carried out in water under transition metal-free conditions. For example, Joshi et al. used TBAHS phase transfer catalyst in a [3+2]-cycloaddition to generate 1,4-diaryl-5-alkyl-1,2,3-triazole derivatives in up to 95% yields and excellent regioselectivity (Scheme 4i) [66]. To reach these good results, elevated temperature and the presence of strong base (KOH) were necessary. The use of triazole-based linkage is also significant in bioconjugation for labeling of biomolecules inside the living cells. The pioneer of bioorthogonal chemistry, Bertozzi, was awarded the Nobel Prize in Chemistry (2023) along with Meldal and Sharpless (click chemistry) [67]. Following the biorthogonal concept, Li et al. have recently described a metal-free, open-air multicomponent reaction of *α*-CF_3_ carbonyls, NaN_3_ and amines to generate 5-amino NH-1,2,3-triazole derivatives selectively (Scheme 4h) [68]. It was also shown how the resulting products are converted into their *N*-2 alkylated derivatives. Bubyrev et al. also introduced two three-component synthetic strategies without using any catalyst or chemical promoter [69]. First, they observed the formation of 1,5-di-substituted-1,2,3-triazoles by mixing *N*-methyl, *N*-phenyl *α*-acetyl- *α*-diazomethane sulfonamide, primary amines and aldehydes.

The reaction occurred in a two-step fashion, and elevated temperature was needed to eliminate sulfur(IV) oxide and *N*-methyl aniline and obtain the desired triazoles. To avoid thermal promotion, the authors developed a second one-step, single purification protocol as well, where the reaction was conducted at room temperature for 18 h, in the presence of 4 Å molecular sieves (Scheme 4g). A further example of metal and organic solvent free synthesis of triazoles was presented by Wan et al. (Scheme 4f) [70]. Readily available *β*-thioenaminones and tosyl azide building blocks were used in the presence of TMEDA (*N,N,N′,N′*-tetramethyl- ethylenediamine) base promoter. No additional catalyst or reagent was needed to achieve the formation of several 5-thiolated 1,2,3-triazoles in good to excellent yields. There are also examples of building alternative, greener metal catalytic systems. Tajbakhsh and Naimi-Jamal described a classic azide-alkyne cycloaddition (AAC) reaction in aqueous media using a Cu@TSC-β-CD (immobilized Cu(I) in thiosemicarbazide-functionalized β-cyclodextrin) nanocatalyst (Scheme 4e) [71]. In the presence of the water soluble and stable catalyst, the desired 1,4-disubstituted-1,2,3-triazoles were obtained in up to 98% yield. The catalyst was reused seven times without significant leaching of Cu(I) and its recovery only required anti-solvent precipitation and filtration. However, the use of copper is often cited as a negative feature of click reactions, due to its cytotoxic effect. Pan and co-workers presented a photocatalytic and metal-free alternative and synthesized various 1,4-disubstituted 1,2,3-triazoles under mild conditions (Scheme 4d) [72]. In the presence of TPPT-Cl (2,4,6-tris(4-chloro-phenyl)pyrylium tetrafluoroborate) photocatalyst, with visible light activation, the corresponding 1,4-disubstituted 1,2,3-triazoles were obtained in moderate yield. The presented protocol shows promising results, but at the same time it still requires further development to match the efficiency of transition metal catalysts. In addition to photochemistry, other non-traditional activation methods have also been adopted to synthesize triazoles [73,74,75,76]. Rodríguez et al. have introduced a microwave-assisted, one-pot multicomponent copper-catalyzed azide-alkyne cycloaddition (CuAAC) followed by a hydrolysis reaction to obtain the corresponding triazoles (Scheme 4c) [77]. As a catalyst, they used copper-based nanoparticles in biorenewable solvents (H_2_O and methanol). Another example of implementing sonochemical activation in cycloaddition reactions has described a catalyst-free synthesis protocol to generate 4-acyl-1,2,3-triazoles and 1,5-disubstituted-1,2,3-triazoles using ultrasound irradiation in an aqueous medium (Scheme 4b) [78]. The protocol is characterized by excellent regioselectivity, scalability, short reaction time and broad substrate scope. Another group has developed a similar water-based ultrasonic method with the exception that a catalyst (1 mol%) was added to the reaction mixture (Scheme 4a) [79]. The multicomponent click reaction, mediated by a Cu(I) complex, afforded 1,4-disubstituted 1,2,3-triazoles in yields up to 93%. In similar approaches, a novel hybrid nano catalyst, silica-tethered cuprous acetophenone thiosemicarbazone (STCATSC) [80] or a silica gel-immobilized [Cu(cdsalMeen)] [81], were used respectively, to promote the synthesis of new 1,2,3-triazoles.

### 2.4. Four-Nitrogen-Containing Heterocycles: Tetrazoles

**Scheme 5:**
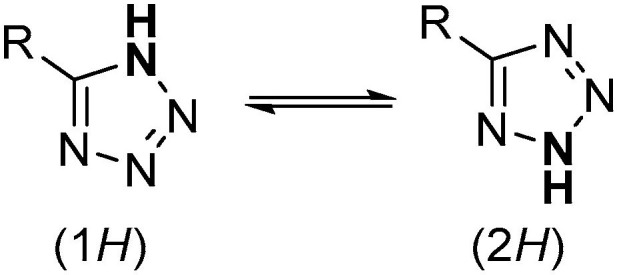
The tautomers of tetrazole derivatives.

Tetrazoles are five-membered heterocycles with four nitrogen atoms in the aromatic ring. They are not natural products; they can only be produced synthetically. Based on how many substituents are attached to the ring, we can distinguish un-, mono-, di- and trisubstituted tetrazoles. The 5-substituted tetrazole is of particular interest because of its similar physical chemical properties to carboxylic acids (mobile H, comparable pKa, similar size) which suggests similar receptor ligand interactions as well. At the same time, they typically have better ADME properties. There are two tautomeric forms of the mono 5-substituted tetrazoles (Scheme 5). Interestingly, while the 2*H*-tautomer is more stable in gas phase, the 1*H*-tautomer is prevalent in solution.

The occurrence of tetrazole moiety in industrially relevant compounds is extensive and has increased dramatically in the last decade. It can be found in photography, imaging chemicals and military applications [82,83], not to mention its significance in medicine and thus the pharmaceutical industry [84,85]. Amongst others, it is the building block of several antibiotics (Cefotiam, Cefmetazole), antihypertensive (Valsartan, Losartan) and antiallergic agents (Pemirolast). Accordingly, there is still a great demand for the development of efficient and sustainable synthesis procedures.

**Scheme 6:**
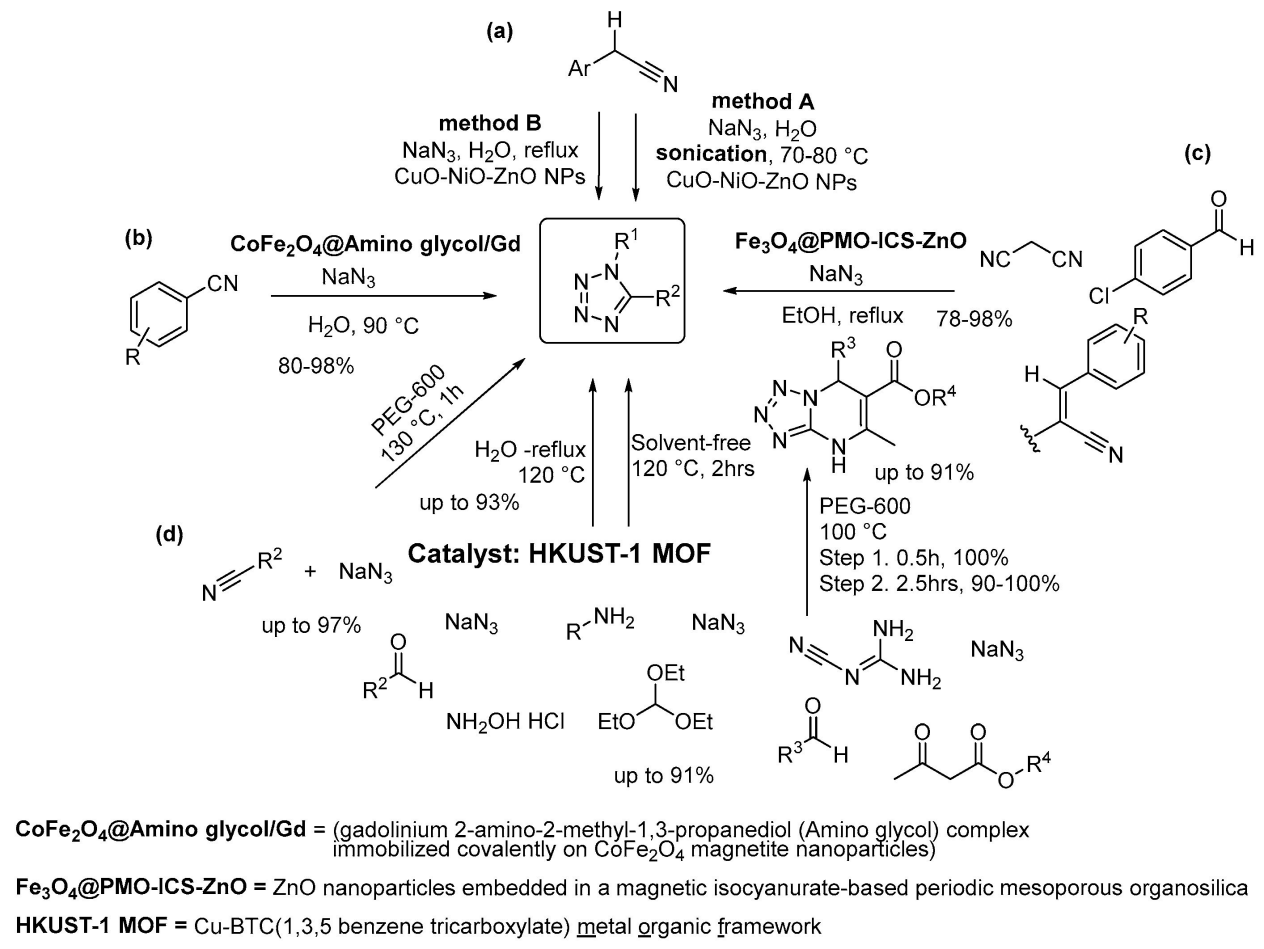
Heterogeneous catalytic tetrazole synthesis protocols.

Heterogeneous synthesis methods have been prominent in the last few years among sustainable protocols for tetrazole synthesis. Scheme 6 illustrates a few highly efficient and selective nanoparticle-based catalyst systems.

Tebyanian and his coworkers have demonstrated a combination ultrasound/nanoparticle catalytic system in regioselective synthesis of 1-aryl-5-amino-1*H*-tetrazoles (Scheme 6a) [86]. The authors tested the CuO–NiO–ZnO mixed metal oxide catalyst in a traditional as well as in an ultrasound-assisted system. Better yield and selectivity were achieved with ultrasounds. The improvement was explained by a probable synergistic effect between the ultrasound irradiation and the nanocatalyst.

In another example, Ghadermazi et al. have developed a CoFe_2_O_4_@amino glycol/Gd nanocomposite to catalyze the oxidation of sulfides and to generate 5-substituted 1*H*-tetrazoles under benign conditions (Scheme 6b) [87]. The corresponding products were obtained in good to excellent yields, and the optimized conditions were tested for broad substrate tolerance. Due to its magnetic nature, the catalyst could be easily removed with the help of an external magnet. Magnetic nanoparticles in general are known to have many favorable properties, such as low toxicity, high surface-to-volume ratio, good thermal stability and high activity, and can be easily modified and dispersed. Another group, Akbari and Naimi-Jamal, have developed an environmentally friendly procedure for cascade condensation and concerted 1,3-cycloaddition reactions catalyzed by magnetic Fe_3_O_4_@PMO–ICS–ZnO nanomaterial (Scheme 6c) [88]. The catalyst retained its activity in five consecutive runs in a water/EtOH mixture under reflux. The 5-substituted-1*H*-tetrazole derivatives were obtained in high to quantitative yields. Lastly, Soroush et al. have demonstrated a new metal organic framework (MOF)-based heterogeneous catalytic system for the multicomponent synthesis of tetrazole derivatives (Scheme 6d) [89]. The hydrothermal technique proved to be the best for preparing the catalyst. In the presence of HKUST-1 MOF, two-, three- and four-component reactions were carried out under mild reaction conditions, using PEG-600, water or solvent-free media. All reactions yielded the desired products with good to excellent yield. Other heterogeneous catalytic protocols include a [3+2] cycloaddition of alkyl nitriles and sodium azide using a silica-anchored copper bis(diacetylcurcumin) 1,2-diamino benzene Schiff base complex with ascorbic acid in a water/i-PrOH (50:50, V/V) medium [90].

**Scheme 7:**
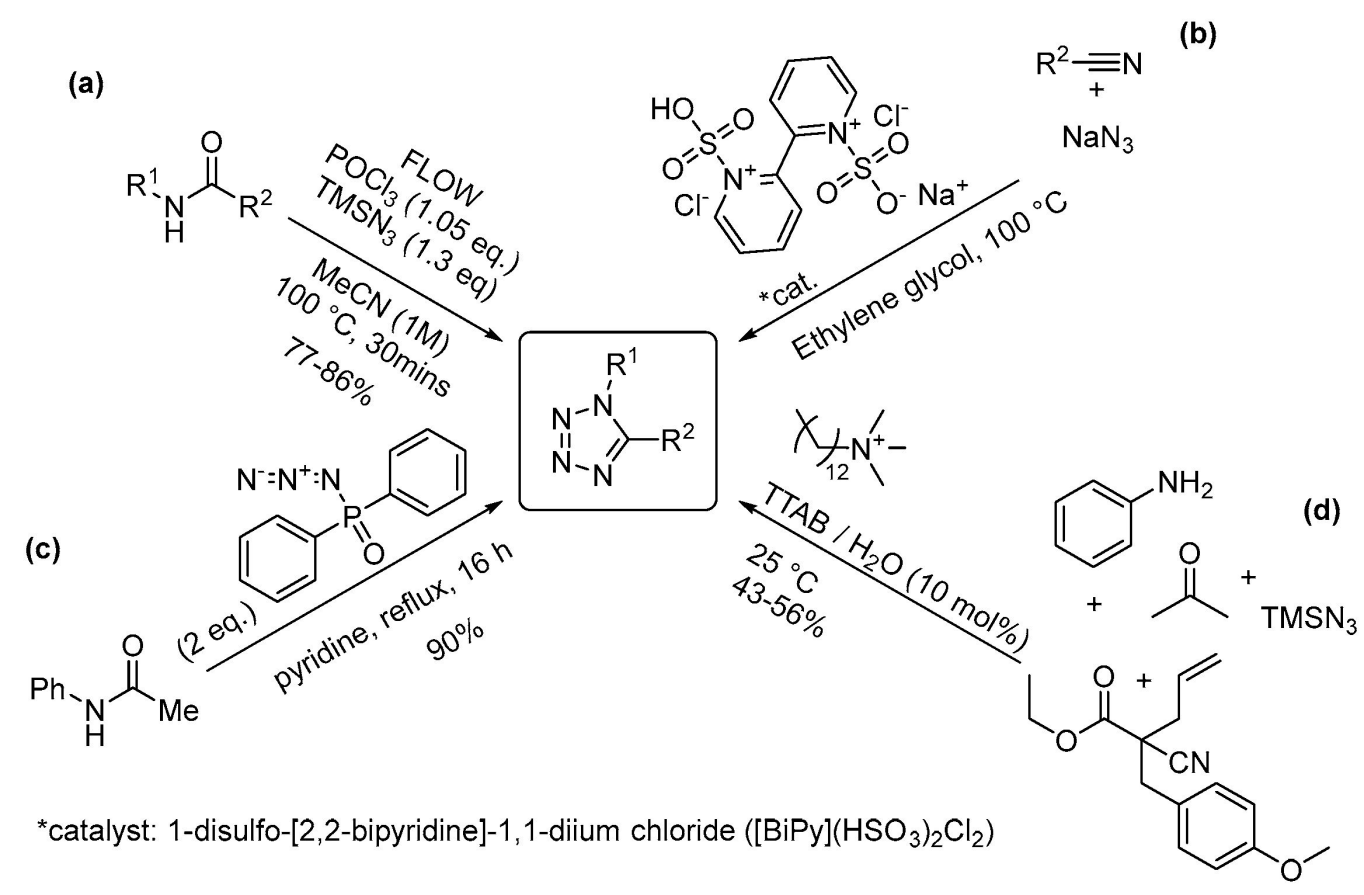
Environmentally benign synthesis methods for tetrazole derivatives.

In addition to heterogeneous catalysis, homogeneous processes have also been applied in recent years as shown in Scheme 7.

An example of using amides as one of the starting materials was illustrated by Kappe’s group in a continuous flow system (Scheme 7a) [91]. The amide was activated by POCl_3_ to imidoyl chloride which in the following step reacts with the azide (TMSN_3_). The reaction reaches full conversion in 10 min and the products were obtained in good yield (77–86%) after recrystallization. No further purification was necessary. Gholizadeh and coworkers have developed a novel protocol for a [2+3] cycloaddition reaction catalyzed by an ionic liquid system to synthesize 5-substituted 1*H*-tetrazole derivatives (Scheme 7b) [92]. Ionic liquids are considered as easily available, non-volatile and non-flammable materials with good thermal and chemical stability.

They are also generally well miscible with both inorganic and organic solvents and reagents. In a short reaction time, the desired products were obtained in very good yields (92–99%) with a wide range of substrates. Ishihara et al. have developed a tetrazole synthesis, starting from amides and working with an alternative azide source [93]. To convert the amides, the authors used diphenyl phosphorazidate (DPPA, (C_6_H_5_O)_2_P(O)N_3_)) or bis(*p*-nitrophenyl) phosphorazidate (*p*-NO_2_DPPA, (*p*-NO_2_C_6_H_4_O)_2_P(O)N_3_)) in the presence of aromatic bases (Scheme 7c). Both DPPA and *p*-NO_2_-DPPA act as an activator of amide-oxygen for elimination and thus an azide source. Mechanistically, what makes this reaction safer compared to the conventional click protocol is that the phosphorus atom stabilizes the azide. This practical and simple protocol is a great example of how toxic and explosive reagents in click reactions can be replaced by safer alternatives. Using water in organic reactions often causes difficulties in terms of solubility. To overcome this challenge, Abdessalam and his coworkers presented a micelle-based Ugi-azide four-component synthesis of 1,5-disubstituted tetrazoles (Scheme 7d) [94]. As starting material, the authors used aldehyde, various amines, isocyanides and trimethylazides in the presence of tetradecyltrimethylammonium bromide (TTAB) with a load of 10 mol% in an aqueous medium. Broad substrate scope was investigated and the corresponding tetrazole derivatives were obtained in moderate yield (43–56%).

## 3. Six-Membered Rings

### 3.1. One-Nitrogen-Containing Heterocycles: Pyridines

**Scheme 8:**
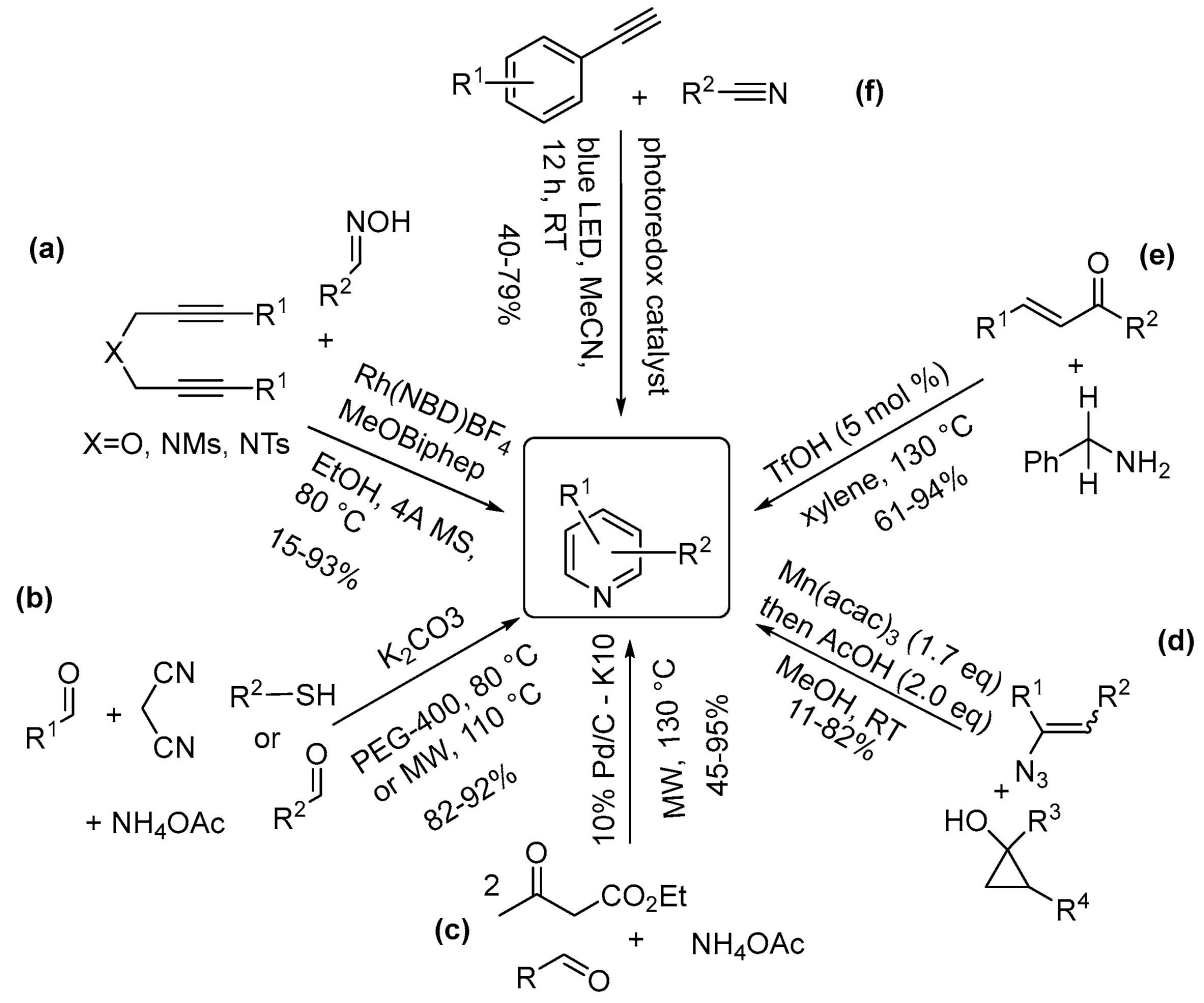
Representative examples of environmentally benign synthesis methods for the preparation of pyridines.

Pyridines are six-membered heterocycles with one N atom in their ring. They are biologically active compounds, and the pyridine scaffold is often used as part of drugs. Thus, they are common building blocks. Their practical utility generated significant interest in their synthesis [95]. Some representative environmentally benign methods are depicted in Scheme 8.

The Rh-catalyzed [2+2+2] cycloaddition of diynes with oximes affords the formation of pyridine derivatives in low to excellent yields (Scheme 8a) [96]. The reaction can be carried out by the preformed oxime or as a multicomponent reaction. Ketoxime acetates also appeared to work in this reaction with aldehydes. FeCl_3_ was used as a water tolerant Lewis acid [97]. These protocols have demonstrated good functional group tolerance and scalability. Similar three- and four component reactions of aryl aldehydes, malonitrile and thiophenols and ammonium acetate yielded a broad variety of substituted pyridines (Scheme 8b) [98]. The reactions were carried out in PEG-400 or methanol as solvent with K_2_CO_3_ on NH_4_OAc as a base catalyst [99]. Solvent-free conditions combined with microwave activation appeared to work sufficiently as well [100,101]. In an enhanced microwave-assisted effort, symmetrically substituted pyridines were prepared by a bifunctional metal–solid acid catalyst via a domino cyclization–aromatization approach [102]. The solid acid catalyzed the cyclization to dihydropyridine and the added Pd promoted the dehydrogenation to pyridines resulting in the aromatic products in moderate to high yields (Scheme 8c). Tetrasubstituted pyridines were prepared by Wang and Chiba using a synthetically diverse approach. The authors used a Mn(III)-assisted addition of vinyl azides and cyclopropanols in a green solvent, methanol at room temperature (Scheme 8d) [103], although the yields showed a great variety from 11–82%. A water tolerant Brønsted acid, triflic acid, was used as a catalyst in a one-pot protocol for the synthesis of pyridines, without the need of harmful oxidizing reagents (Scheme 8e) [104]. The reaction occurred via a tandem reverse aldol reaction/condensation/cyclization /aromatization sequence with enones and primary amines. The excellent functional group tolerance, air as a naturally abundant and green oxidant and the water tolerant acid catalyst are the major green advantages of the procedure. Finally, visible-light-promoted processes have also found use in the synthesis of pyridines. A blue LED irradiation-initiated [2+2+2] cyclization of alkynes and nitriles led to the successful synthesis of a broad variety of pyridines (Scheme 8e) [105]. The process was catalyzed by a photoredox catalyst and occurred with excellent functional group tolerance in moderate to good yields.

### 3.2. Two-Nitrogen-Containing Heterocycles: Pyrimidines, Pyrazines

Pyrimidines are six membered ring heterocyclic compounds containing two nitrogen atoms. They make up an important group of compounds and have thus attracted significant attention, and their synthesis development is still at the forefront of organic synthesis [106,107,108].

**Scheme 9:**
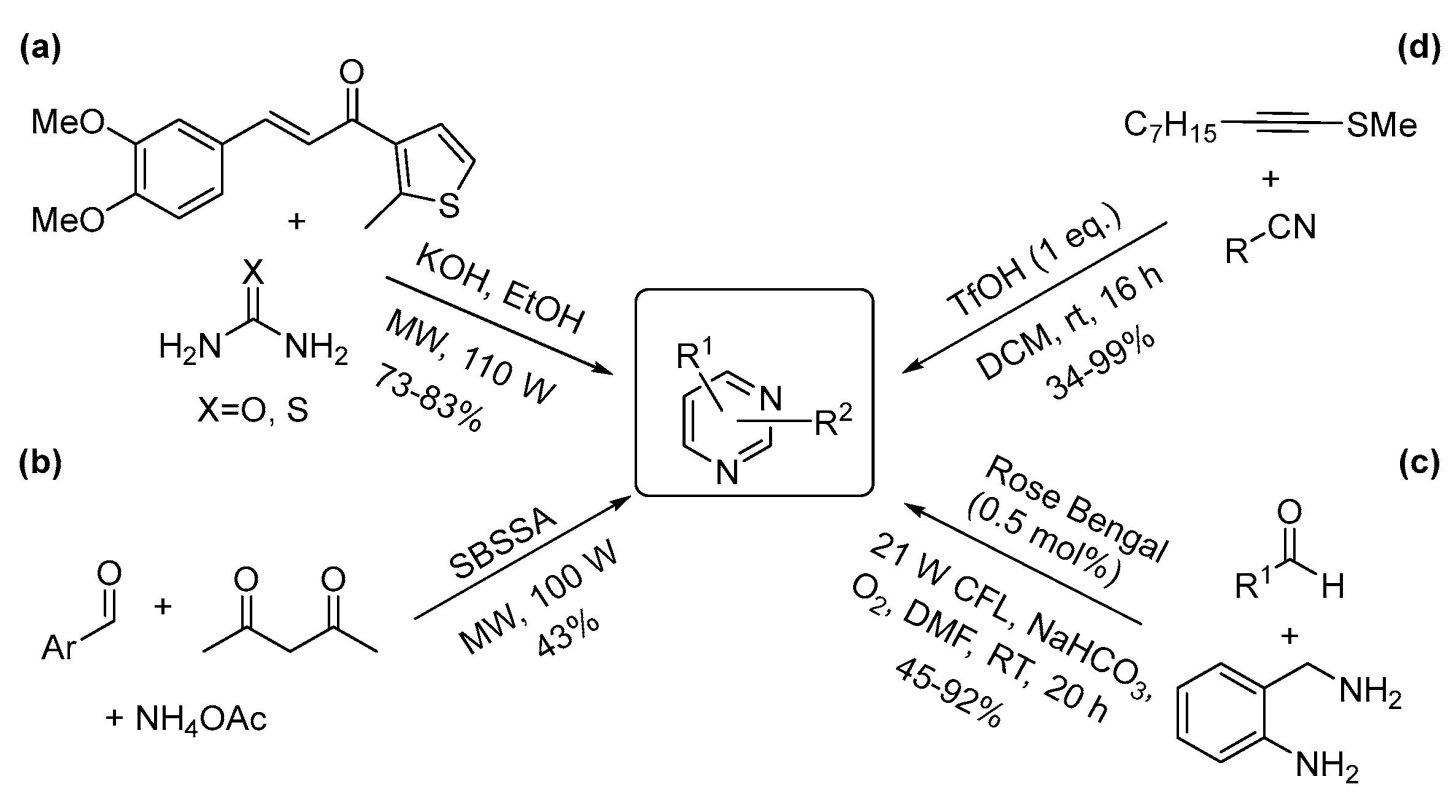
Representative examples of environmentally benign synthesis methods for the preparation of pyrimidines.

**Scheme 10:**
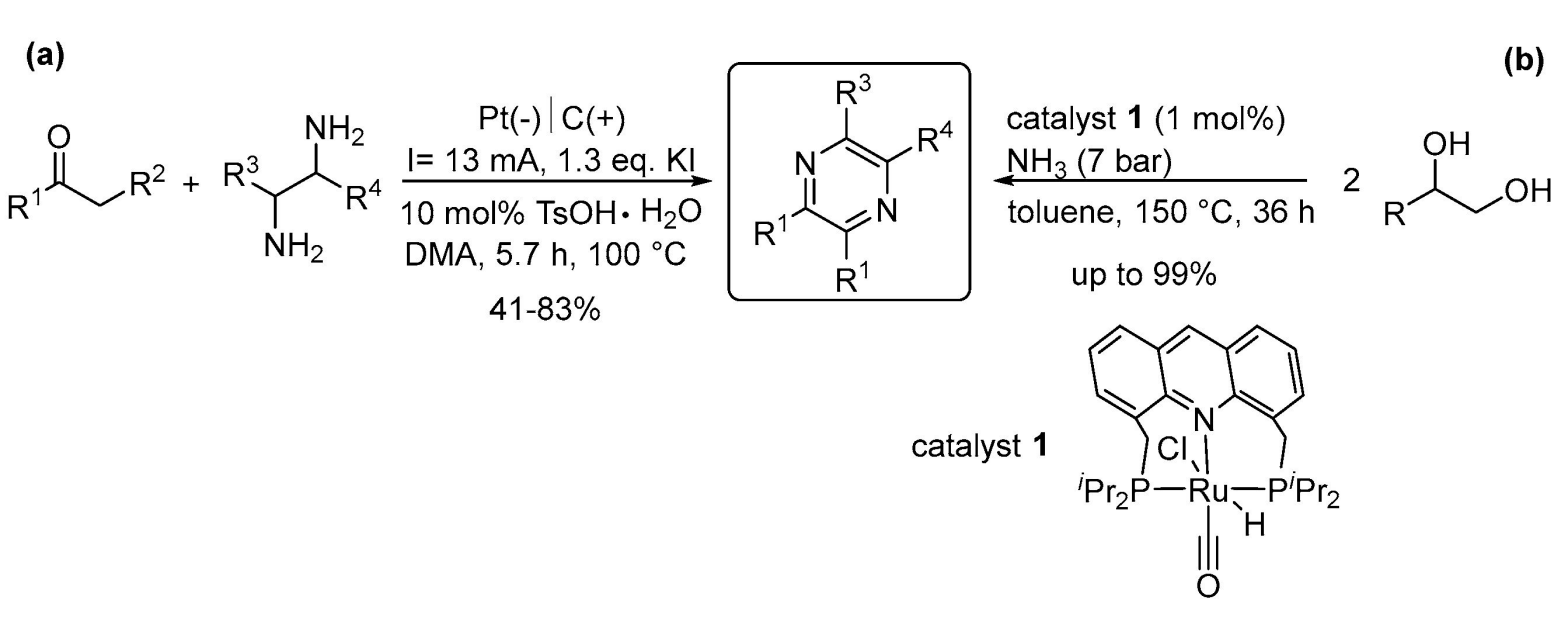
Representative examples for the environmentally benign synthesis of pyrazines by an electrochemical and a catalytic method.

Well-known and widely available starting materials such as chalcones with urea and thiourea were used for the preparation of pyrimidines (Scheme 9a) [109]. Microwave irradiation was used to activate the process that provided the substituted pyrimidines in good yields under green conditions e.g., using ethanol, a sustainable and green solvent. Other similar applications include the use of guanidine nitrate instead of urea derivatives [110], or a CuI and base-catalyzed protocol [111]. The synthesis of pyrimidines was also accomplished by a three-component reaction that was catalyzed by a silica-bound S-sulfonic acid (SBSSA) (Scheme 9b) [112]. The microwave-assisted protocol involved a solvent-free reaction, in <1 min reaction times. Urea could be used to replace ammonium acetate; however, the major limitation of the reaction is the moderate yield (43%) and that the non-aromatic tetrahydropyrimidines were isolated. Condensed pyrimidines were prepared by a visible-light-LED-initiated cyclization of primary amines and aldehydes in moderate to excellent yields under mild conditions (Scheme 9c) [113]. The reaction was catalyzed by 0.5% of Rose Bengal as a photocatalyst and was carried out in air at ambient temperature. Using DMF as a solvent somewhat decreases the green synthetic value of the protocol. The water tolerant triflic acid has been applied as a catalyst for the synthesis of pyrimidines as well. The regioselective combination of alkynes and nitriles was catalyzed by Brønsted superacid, TfOH providing the pyrimidines with high yield and selectivity (Scheme 9d) [114]. An extensive list of diverse and readily available nitriles was applied in the cycloaddition under a simple protocol and mild conditions, although the use of dichloromethane is not desirable. Pyrazines contain their two nitrogen atoms in a 1,4-position. Their synthesis is highly attractive due to their broad-spectrum biological activity. Among the several examples we highlight an electrochemical process and a catalytic process. The electrochemical dehydrogenative [4+2] annulation of a broad variety of commercially available ketones and diamines resulted in the formation of pyrazines in moderate to high yields (Scheme 10a) [115]. The electrochemical oxidation combined with Brønsted acid catalysis led to the target products. In another example, a Ru-pincer complex was used as a catalyst in the synthesis of pyrazines from diols. The oxidative coupling of 1,2-diols and ammonia as the nitrogen source resulted in the formation of pyrazines in nearly quantitative yields (Scheme 10b) [116]. Although the protocol has some drawbacks (using toluene, and long reaction time at high temperature) the use of NH_3_, the catalyst, the high atom economy and low amount of nontoxic waste are clear green benefits.

### 3.3. Three- and Four- Nitrogen-Containing Heterocycles: Triazines and Tetrazines

Both triazine and tetrazine molecular scaffolds are widely used in biorthogonal chemistry as cell labeling, diagnostic, coordination, drug release or live cell imaging agents. Due to their broad application, they attracted extensive attention in the last few years [117,118,119,120]. The Pinner reaction is considered the classic tetrazine synthesis when the reaction of activated nitriles with hydrazines occurs followed by an oxidation step. There are highly efficient transition metal-based tetrazine synthesis protocols, mostly for biorthogonal chemistry applications [121,122].

**Scheme 11:**
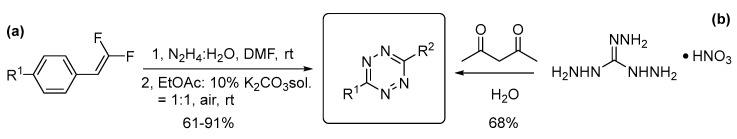
Environmentally benign protocols to synthesize 1,2,4,5-tetrazoles.

Recently, the replacement of old synthetic methods with sustainable processes has been gaining ground. Fang et al., have presented a [3+3] addition reaction starting from *gem*-difluoroalkenes to synthesize both symmetric and asymmetric 3,6-disubstituted 1,2,4,5-tetrazine derivatives under benign conditions (Scheme 11a) [123]. The reaction was carried out in an aqueous medium at room temperature under air, which also serves as an oxidant replacing the commonly used toxic nitric acid. The gram scale of the reaction was presented, and the corresponding products were obtained with a moderate to good yield (61–91%). Another green synthesis protocol was developed to generate secondary explosive materials, 3,6-bis[2-(4,6-diazido-1,3,5-triazin-2-yl) -hydrazinyl]-1,2,4,5-tetrazine and 3,6-bis-[2-(4,6-diazido-1,3,5-triazin-2-ly)-diazenyl]- 1,2,4,5-tetrazine (Scheme 11b) [124]. The major goal was to replace Pb(N_3_)_2_ and generate a metal-free alternative. Due to their high nitrogen content, tetrazines often serve as building blocks for the preparation of high-energy, low-sensitivity explosives.

**Scheme 12:**
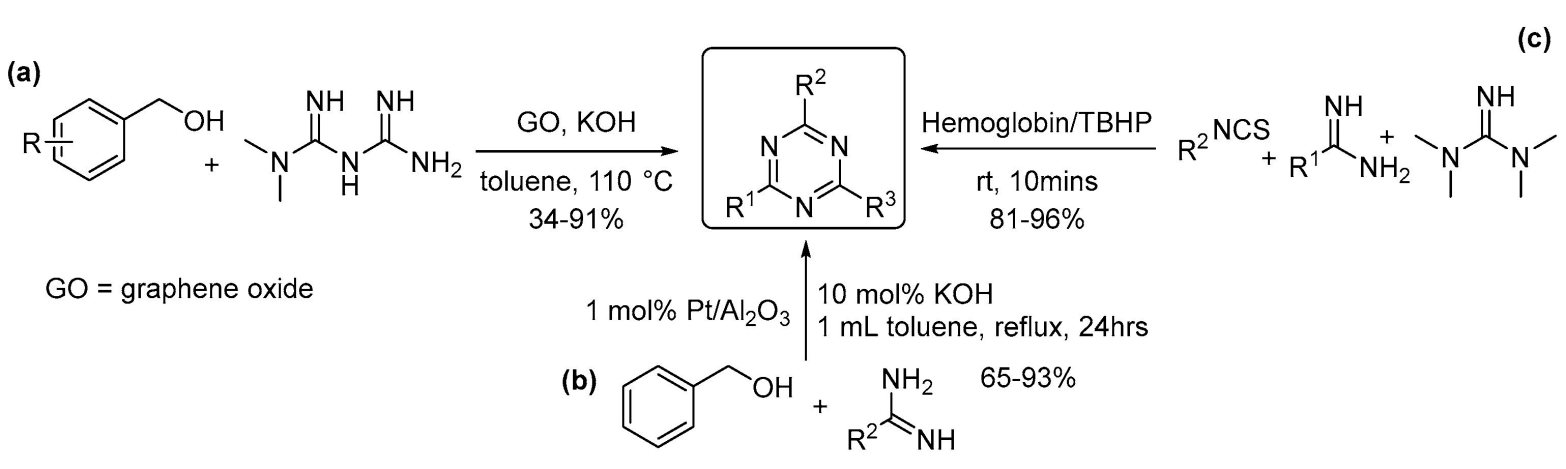
Sustainable, green protocols to synthesize 1,3,5 triazine derivatives.

Triazines are six-membered aromatic heterocyclic compounds containing three nitrogen atoms. One of the most common isomers is 1,3,5-triazine, which exhibits several biological activities, such as antimalarial and anti-HIV activity. A novel graphene oxide-based catalyst was developed for the synthesis of triazines (Scheme 12a) [125]. The catalyst was used at moderate loading (10 mol%) and proved to be reusable in six runs without significant loss of activity. At the end of the reaction, the desired products were obtained in moderate to good yields up to 91%. In another example, Poly et al. demonstrated the use of primary alcohols and amidines as molecular building blocks for triazine synthesis by applying alumina-supported Pt (Pt/Al_2_O_3_) nanoparticle catalyst (Scheme 12b) [126]. The acceptorless dehydrogenative coupling reaction was carried out in one pot with high atom economy and good yields of up to 93%. Separation of the catalyst was easy after reuse and no extra oxidants were needed to complete the reaction. Biomolecules have also been reported to catalyze the synthesis of triazines. Wang et al. introduced an efficient asymmetric synthesis of 1,3,5-triazines in the presence of hemoglobin (heme concentration: 0.05 mol%) and *tert*-butyl hydroperoxide (TBHP), using isothiocyanate, amidines and 1,1,3,3-tetramethylguanidine [127]. The presented procedure resulted in high yields (81–96%) in a short reaction time at room temperature (Scheme 12c).

**Scheme 13:**
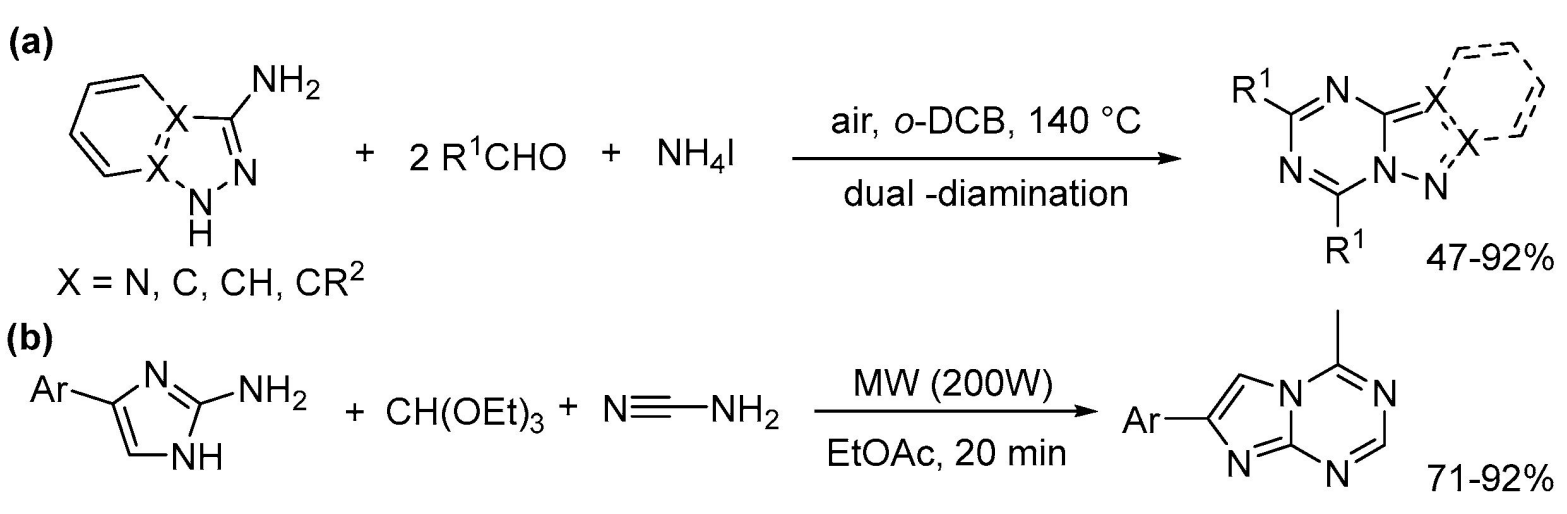
Green protocols for the synthesis of purine isosteres.

Like most heterocycles, the triazines are often present in fused ring systems. Purine is one of the most biologically relevant fused heterocycles as one of the fundamental building blocks of DNA and RNA. Therefore, the synthesis of its isosteres is of great interest due to their therapeutic potential. Scheme 13 shows two examples of the synthesis of pharmaceutically relevant derivatives. The first example illustrates an open-air dual-diamination annulation to generate 5-aza-9-deazapurine derivatives (Scheme 13a) [128]. Commercially available aromatic aldehydes and aminoazoles afforded *N*-azolo amidines in the first step which then reacted with ammonium iodide to yield the corresponding fused triazines in good yield, up to 92%. No catalyst or further additives were required. As another example, a novel efficient synthesis of 5-aza-7-deaza-adenine scaffold was developed using a catalyst-free, microwave-assisted multicomponent reaction of triethyl orthoformate and cyanamide (Scheme 13b) [129]. The protocol afforded the desired 4-aminoimidazo[1,2-a][1,3,5]triazine derivatives in good yield and selectivity, in short reactions with good scalability and reproducibility.

## 4. One-Nitrogen-Containing Condensed Heterocycles

Condensed heterocycles contain multiple rings and commonly serve as the backbone of natural products such as alkaloids, amino acids or nucleic acids. Their structures inspired extensive synthetic efforts to build bioactive compounds that could be applied as drug candidates. Several strategies outlined above already involved the preparation of condensed heterocycles; however, here the emphasis will be on these specific structures.

### 4.1. Indoles

**Scheme 14:**
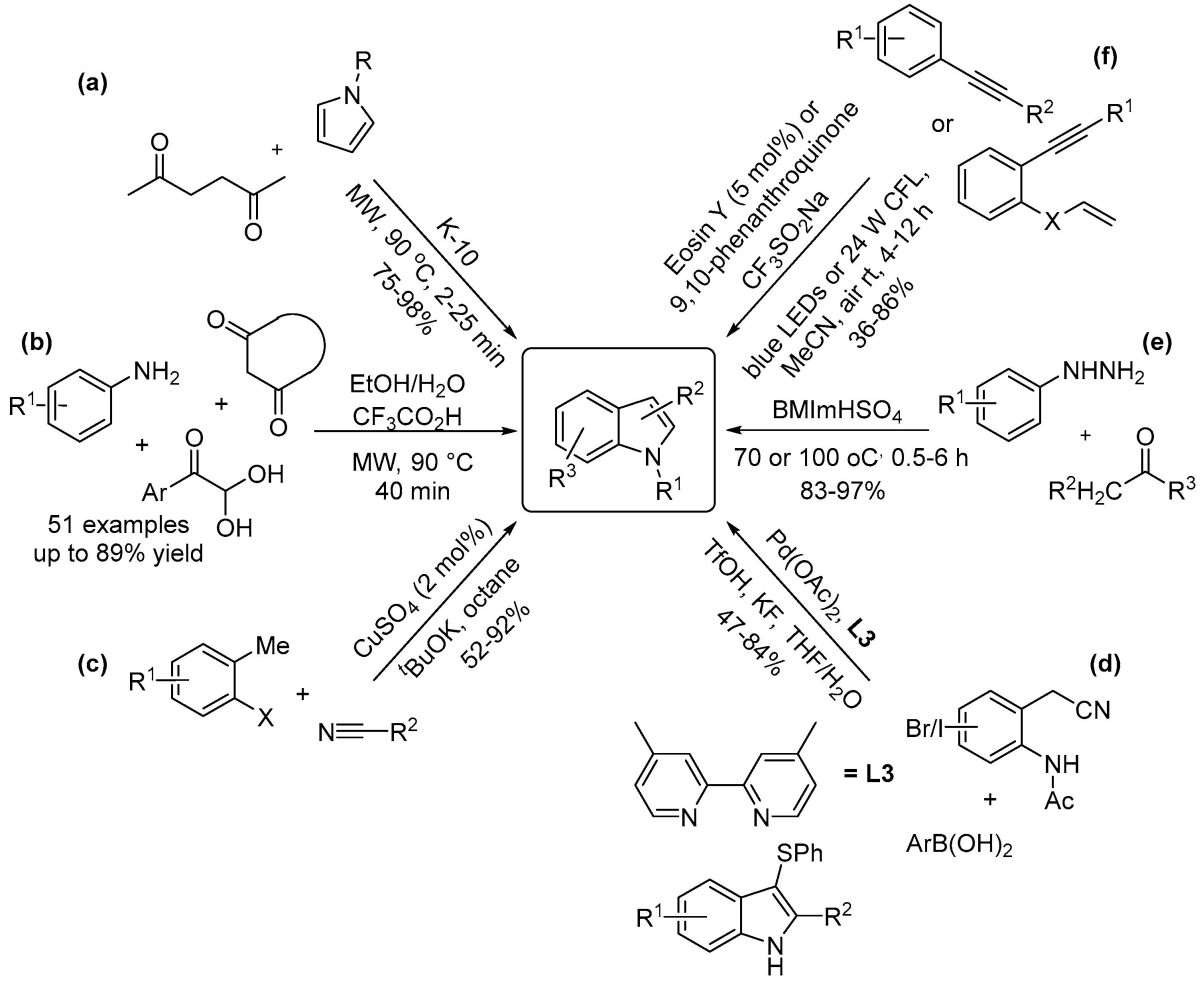
Representative green protocols for the synthesis of indoles.

Indoles are one of the most frequently used and synthesized condensed heterocycles. Representative, environmentally benign, synthesis protocols are depicted in Scheme 14.

Pyrroles readily formed indoles in a reaction with 1,4-dicarbonyl compounds. The solvent-free microwave-assisted reaction was catalyzed by K-10 montmorillonite (Scheme 14a) [17]. K-10 is a solid acid that is also an effective microwave absorber, serving as the medium for the process. The protocol has many green advantages: recyclable solid catalyst, solvent-free conditions, microwave heating, high atom economy and excellent yields, short reactions and a small amount of nontoxic waste (water). Another report described a microwave-assisted catalytic method for the synthesis of indole derivatives from anilines, arylglyoxal monohydrates and cyclic 1,3-dicarbonyl compounds (Scheme 14b) [17]. The reactions occurred in short times in green solvents with high yields and regioselectivity. A simple CuSO_4_-catalyzed carbanion-radical redox relay was reported by Shan et al. for the synthesis of N-H indoles (Scheme 14c) [130]. This process could be applied in large-scale preparations as it applies inexpensive reagents. The indole core can also be synthesized by a novel addition/cyclization of 2-(2-aminoaryl)acetonitriles with arylboronic acids catalyzed by a Pd complex (Scheme 14d) [131]. This protocol tolerates a broad range of functional groups and occurs with high selectivity. Ionic liquids (ILs), proposed green solvents, have been used for the preparation of indoles via a modified Fischer indole synthesis providing excellent yields (Scheme 14e) [132,133]. The IL-based methodology occurs without additional solvents and the ILs are generally reusable without any loss in their activity. The emerging, visible-light-promoted activation was also applied for the synthesis of indoles. Using different photoredox catalysts, Eosin Y or 9,10-phenanthrequinone, indoles were synthesized by the vicinal thioamination of alkynes or an intramolecular cyclization of alkynes with alkenes in moderate to good yields under mild conditions (Scheme 14f) [134,135].

### 4.2. Indolizines

**Scheme 15:**
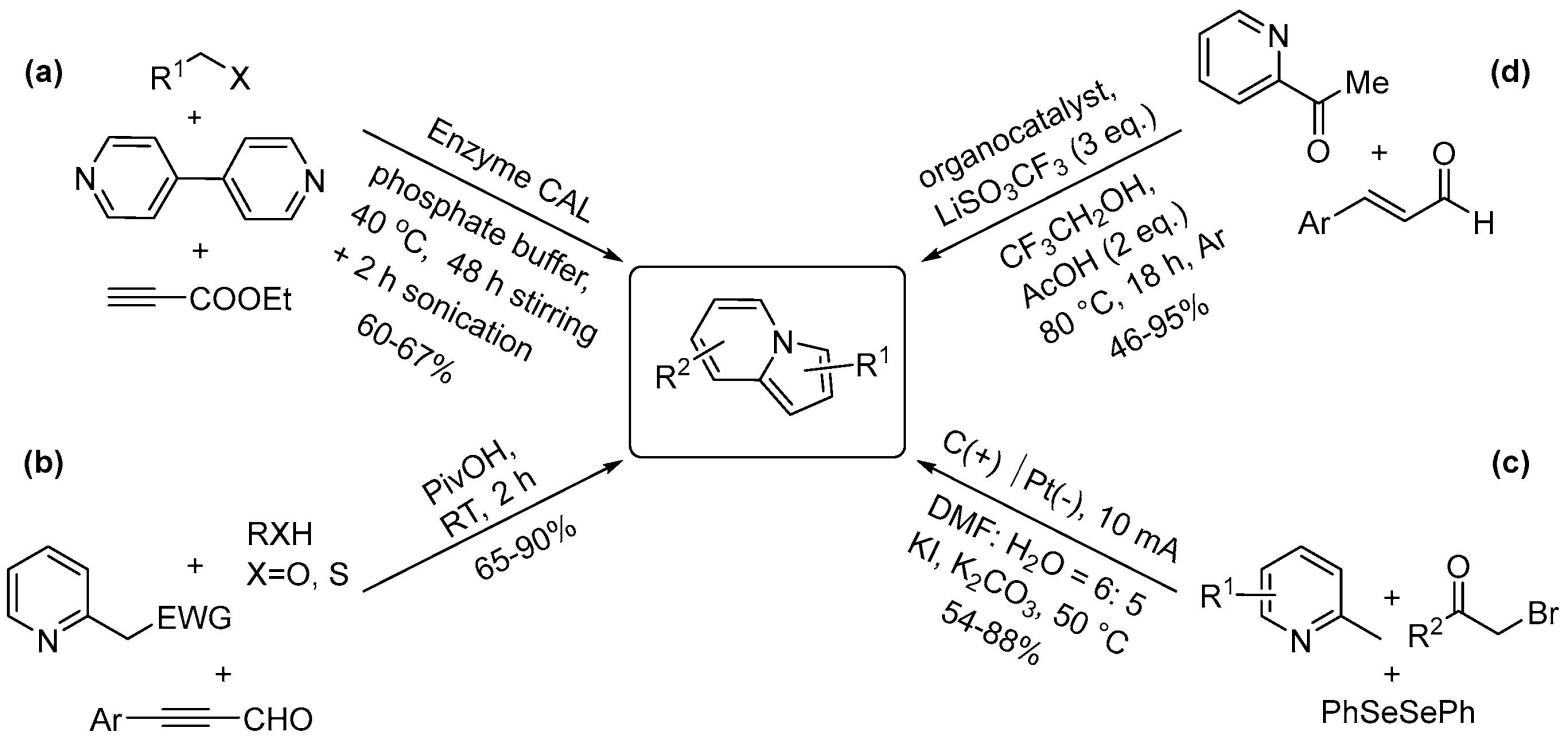
Representative green protocols for the synthesis of indolizines.

Indolizines, a group of indole analogs, have the N atom positioned to the annulation position between the two rings [136]. Their synthesis can be carried out by cycloadditions of 4,4′-bipyridine, halide derivatives and ethyl propiolate using a biocatalytic method [137]. The enzyme *Candida antarctica* lipase (CAL) showed the best performance in the reaction. (Scheme 15a). The protocol carries many green advantages: it is biocatalytic, the products form in good yields and high purity and it occurs in water under mild conditions. Another multicomponent reaction using similar starting materials, such as 2-(pyridin-2-yl) acetates, ynals and alcohols or thiols was catalyzed by pivalic acid under mild and solvent-free conditions (Scheme 15b) [138]. A similar organocatalytic process was also reported for the synthesis of a broad variety of indolizines (Scheme 15d) [139]. Electrochemical activation was also found to be effective for the preparation of indolizines from 2-methylpyridines, α-bromoketones and diselenides (Scheme 15c) [140], without the use of transition metal catalyst or external oxidant.

### 4.3. Quinolines, Isoquinolines

**Scheme 16:**
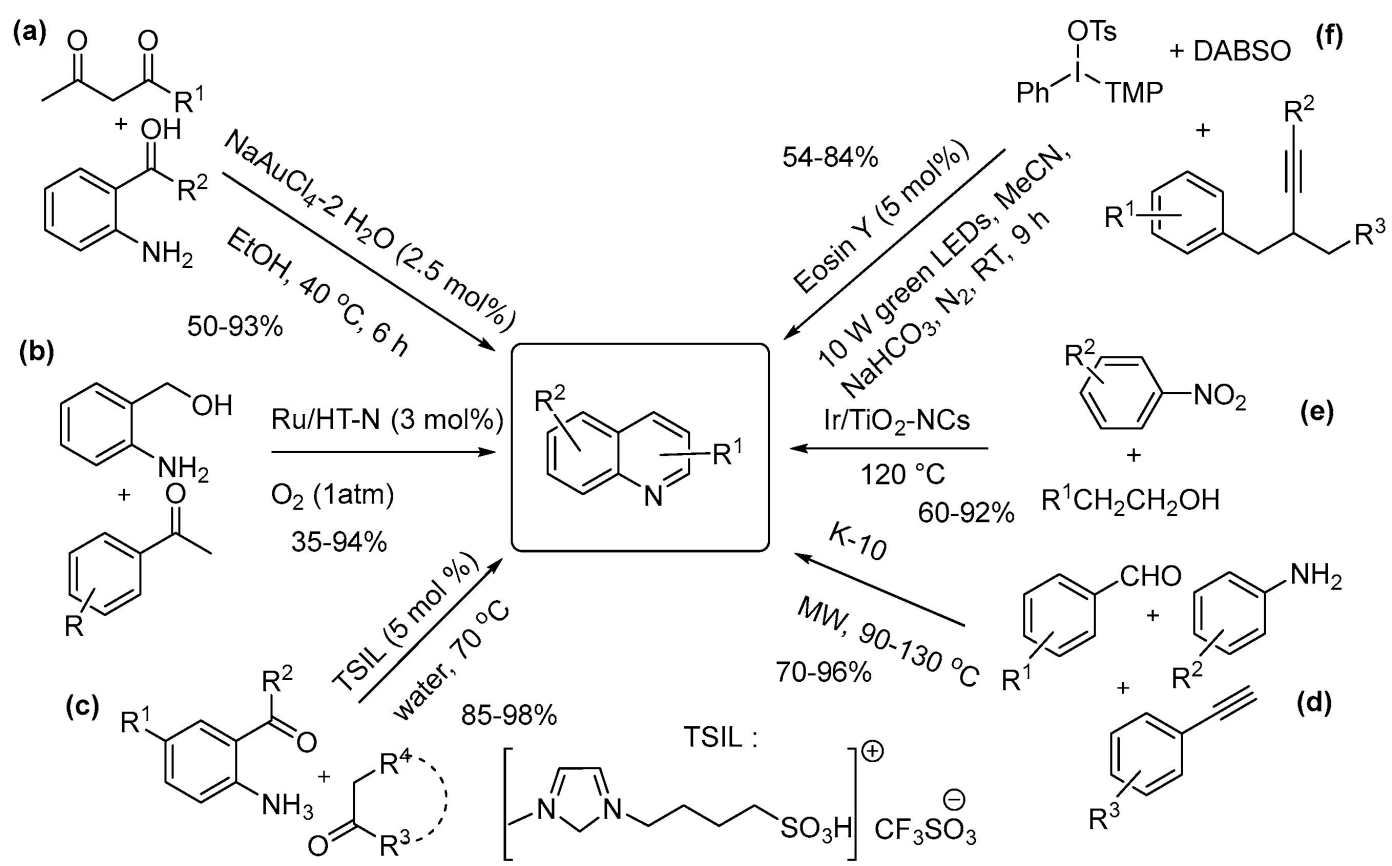
Representative green protocols for the synthesis of quinolines.

The quinoline and isoquinoline skeletons are frequent core units in many bioactive natural products, most prominently alkaloids. Thus, the development of green synthetic protocols is at the forefront of organic synthesis research. A representative group of environmentally benign processes for the synthesis of quinolines are depicted in Scheme 16.

Gold has gained significant attention as a catalytic material [141]. A gold catalyst has been applied in a modified Friedländer synthesis [142] of quinolines, to make the old protocol comply with current safety and environmental standards. The Au (III)-based catalyst promoted the condensation/annulation pathway under mild conditions using 2-amino acetophenones and 1,3-dicarbonyl compounds (Scheme 16a) [143]. In a heterogeneous catalytic approach Ru-grafted hydrotalcite (HT) [144] catalyzed the reaction of 2-aminobenzyl alcohol with carbonyl compounds (Scheme 16b) [145]. Ru-HT was described as a bifunctional catalyst, the basic HT catalyzed the aldol reaction and Ru promoted the oxidative aromatization. Ionic liquids have also been applied for the synthesis of quinolones. The reusable SO_3_H-functionalized alkyl-imidazole, a water tolerant acid, efficiently catalyzed the cyclization of 2-aminoaryl ketones and β-ketoesters/ketones in an aqueous medium in high yields (Scheme 16c) [146]. As a green feature, the products precipitated from the medium and were isolated by a simple filtration thus avoiding solvent demanding purification. In another solid-acid-catalyzed procedure, the recyclable K-10 montmorillonite, was applied in a microwave-assisted solid phase multicomponent reaction (MCR). The reaction of anilines, benzaldehydes and terminal phenylacetylenes yielded 2,4-disubstituted quinolines (Scheme 16d) [147]. K-10 also catalyzed another MCR to provide quinolines [148]. A bifunctional metal–solid acid catalyst, Ir/TiO_2_-NCs (nanoclusters), catalyzed the reaction of nitroarenes and aliphatic alcohols. The reduction–condensation–dehydrogenation pathway provided the quinolines in moderate to excellent yields (Scheme 16e) [149]. As described above the visible-light-promoted photoredox catalysis is attracting much attention in green synthesis. The Eosin Y-catalyzed, green LEDs irradiation-promoted reaction of N-propargyl aryl amines, diaryliodonium salts and sulfur dioxide was carried out at room temperature providing the quinolines in moderate to good yields (Scheme 16f) [150].

**Scheme 17:**
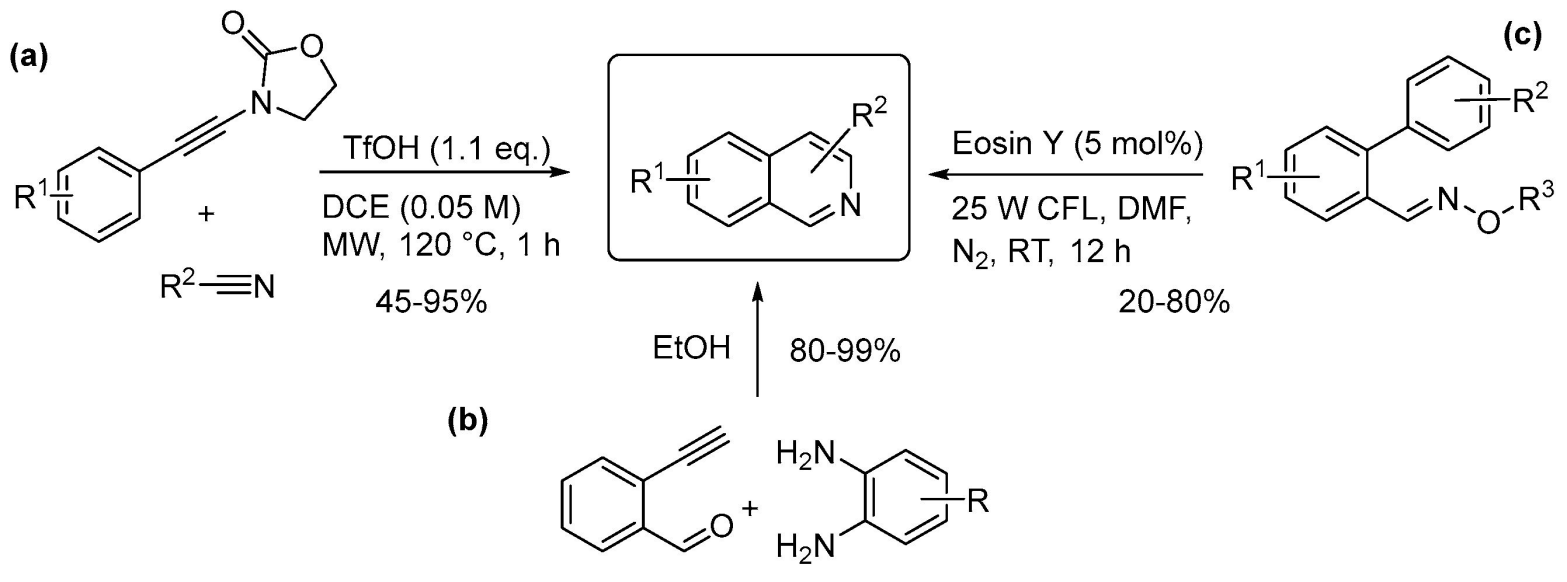
Synthesis of isoquinolines by environmentally benign processes.

Similar protocols have been explored for the synthesis of isoquinolines as well. The water-tolerant Brønsted superacid, triflic acid (TfOH), efficiently catalyzed the regioselective intermolecular cycloaddition of ynamides-alkynes and nitriles (Scheme 17a) [103]. The protocol affords high yields to synthesize a broad variety of isoquinolines from readily available nitriles as the C–N sources. The high atom economy, microwave activation, short reaction times and the water tolerant acid catalyst are all green advantages, although dichloromethane is an undesirable solvent. Another report described the catalyst-free synthesis of benzimidazo[2,1-a]isoquinolines by the coupling of 2-ethynylbenzaldehyde with *ortho*-phenylenediamines. The reactions occurred in ethanol, a not only green but renewable solvent at room temperature (Scheme 17b) [151] generating high yields and minimal environmental impact. Similarly to 4.3.1.f, eosin Y was found to be an efficient photoredox catalyst for the cyclization of O-2,4-dinitrophenyloximes to produce isoquinolines as well (Scheme 17c) [152].

### 4.4. Carbazoles

**Scheme 18:**
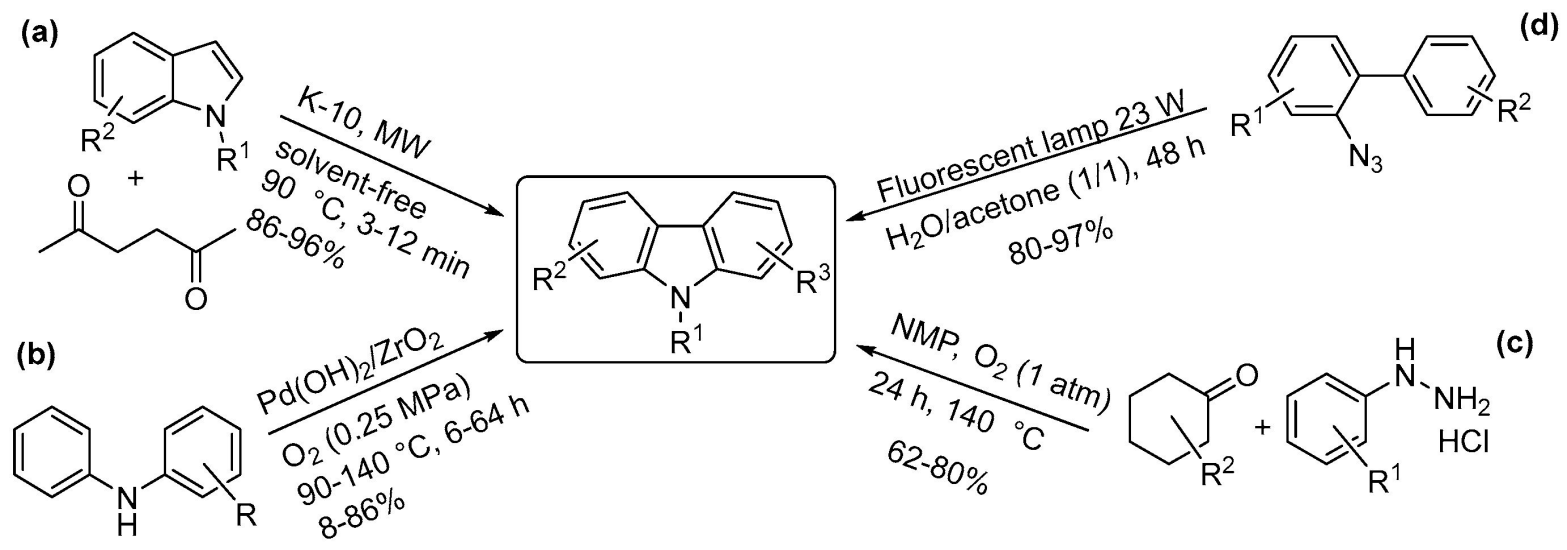
Synthesis of carbazoles by green processes (NMP-N-methyl-pyrrolidone).

Carbazoles are indole derivatives having an additional aromatic ring on both sides of the central pyrrole. Similarly to the above applications, K-10 montmorillonite was found to be effective for the synthesis of substituted carbazoles as well [153]. The microwave-assisted heterogeneous catalytic method resulted in high yields and excellent selectivities under very short reaction times (Scheme 18a). The protocol has high atom economy combined with excellent yields. It is a solvent-free reaction with a solid catalyst and minimal energy consumption, and it is nearly waste-free, producing water as a byproduct. The Pd-catalyzed oxidative intramolecular coupling of diarylamines also led to the formation of carbazoles. The process was catalyzed by a ZrO_2_-supported Pd(OH)_2_ (Pd(OH)_2_/ZrO_2_) with air as the oxidant (Scheme 18b) [154,155]. The synthesis of carbazoles was achieved in a catalyst-free reaction from cyclohexanones and arylhydrazine hydrochlorides in moderate to good yield (Scheme 18c) [156]. The catalyst-free nature and the use of molecular oxygen as an oxidant are the main green advantages of the protocol. A visible-light-activated process successfully cyclized 2-azidobiphenyls to carbazoles in high to excellent yields (Scheme 18d) [157]. This high atom economy/high yielding process occurs in water and produces nitrogen as the only byproduct.

### 4.5. N and Other Heteroatom

When designing the synthesis of fused heteroarenes, one of the first things to decide is which ring should be formed first. Baran et al. gave the following guidelines in an extensive literature review [158]. In essence, it always leaves the easier synthesis for later. For example, if there is a five and a six membered ring fused together, one should start with the six membered ring as a building block and then annulate the five membered one, mainly because in most cases the substitution of the six-membered ring system is easier, as is the synthesis of the five-membered rings. Therefore, it is recommended to first place the necessary functional groups on the six-membered ring for the sequential synthesis of the five-membered ring. Furthermore, if one of the rings in the fused ring system contains multiple heteroatoms, that ring should be synthesized later. It is rooted in the general observation that the synthesis of heteroarenes containing more heteroatoms is easier than the synthesis of heteroarenes with fewer. If each ring contains several heteroatoms, the position of the heteroatoms is decisive. Retrosynthetically, one should first cleave the ring with successive heteroatoms.

**Figure 2:**
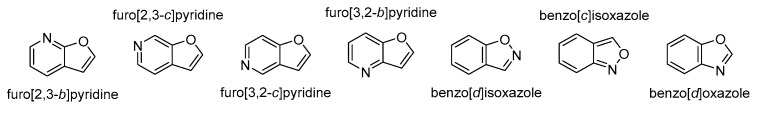
Oxygen-containing *N*-heterocycle isomers.

Here, our goal is to give a representative account of recent developments in green and environmentally benign procedures for synthesis of condensed heterocycles containing one nitrogen atom and another heteroatom (e.g., O, S or Se). The examples were grouped based on the extra heteroatom atom and their position relative to the nitrogen atom. The first group illustrates the oxygen-containing condensed heterocycles (Figure 2).

**Scheme 19:**
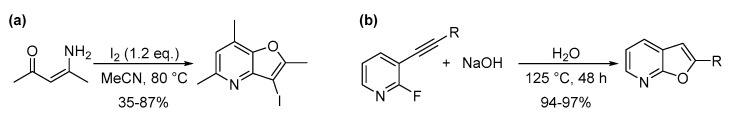
A tandem metal-free cyclization of enaminones in the presence of iodine. Reaction of electrophilic 2-fluorophenylacetylene and sodium hydroxide in an aqueous medium.

Huang et al. presented a one-pot synthesis of substituted furopyridines. (Scheme 19a) [159]. Simple enaminone amides underwent an iodine-mediated and metal-free oxidative tandem cyclization reaction to generate the corresponding furopyridine derivatives in moderate to good yield (35–87%). Furopyridines show biological activity against HIV protease inhibitor, therefore their green and benign synthesis is of great interest. Another synthesis protocol was presented by Katz et al. for generating furo[2,3-b]pyridine derivatives (Scheme 19b) [160]. Reacting the electrophilic 2- fluorophenylacetylene with sodium hydroxide in an aqueous medium gave the desired furopyridine derivative with an excellent yield (94–97%), but it should be noted that excessive substrate tolerance was not investigated. Further acetylene-activated S_N_Ar/intramolecular cyclizations in the synthesis of indole and benzofuran derivatives were also discussed.

**Scheme 20:**
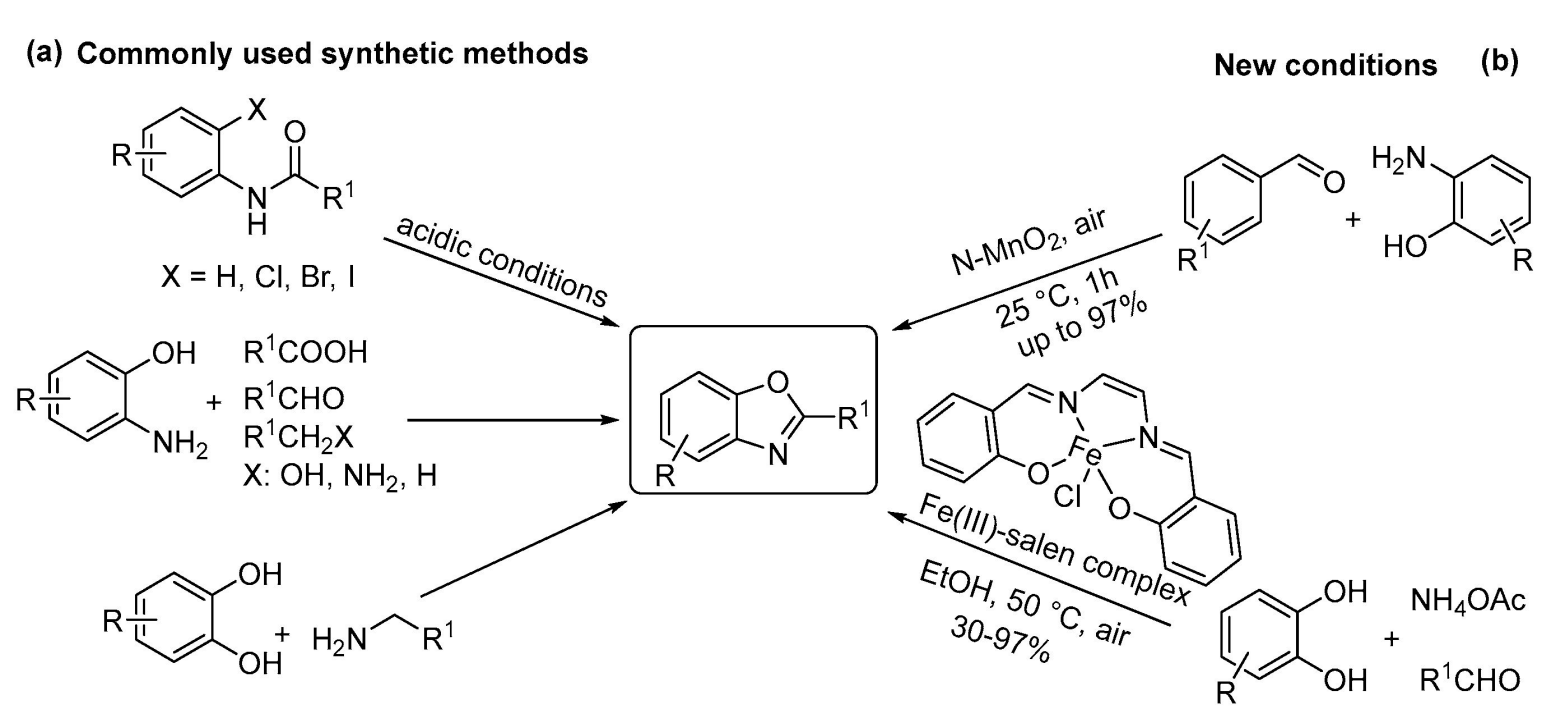
Traditional and novel, environmentally friendly synthetic protocols for generating benzo[d]oxazole derivatives.

Benzoxazole is one of the most widely used isomers of *N*-containing condensed heterocycles. Over the years several procedures have been demonstrated for its synthesis (Scheme 20a) [161]. Recently, Han and Ke have presented a highly efficient, nitrogen-doped manganese dioxide (N-MnO_2_)-catalyzed controllable reaction of 2-amino-phenols and carboxylic acid derivatives for generating benzoxazoles (Scheme 20b) [162,163,164,165]. The procedure was carried out at room temperature, and the benzoxazole was obtained with >99% yield and up to >87% for additional benzoxazole derivatives. No significant decay in catalytic activity was observed during 10 catalytic cycles. The introduction of nitrogen into the defect and the coordinatively unsaturated Mn sites proved to be crucial for the catalytic activity. Sharghi et al. presented a one-pot multicomponent reaction for the synthesis of benzoxazole in the presence of Fe(III)-salen complex (Scheme 20b) [166]. The use of catechols, ammonium acetate and aldehydes as starting materials under mild reaction conditions resulted in the desired benzoxazole derivatives in a short time and with moderate to excellent yield (30–97%). Although the catalysts of both reactions contained metal, the processes yielded the desired product with excellent results, and the catalysts were easily separated at the end of the process.

**Figure 3:**
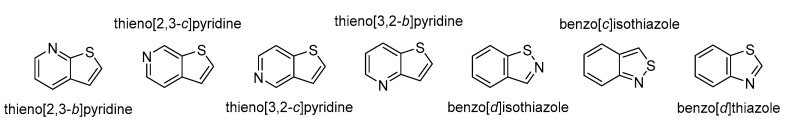
Sulfur-containing N-heterocycles.

After the oxygen-containing condensed 1N containing heterocycles, we continue with the discussion of the synthesis of sulfur containing heterocycles (Figure 3).

**Scheme 21:**
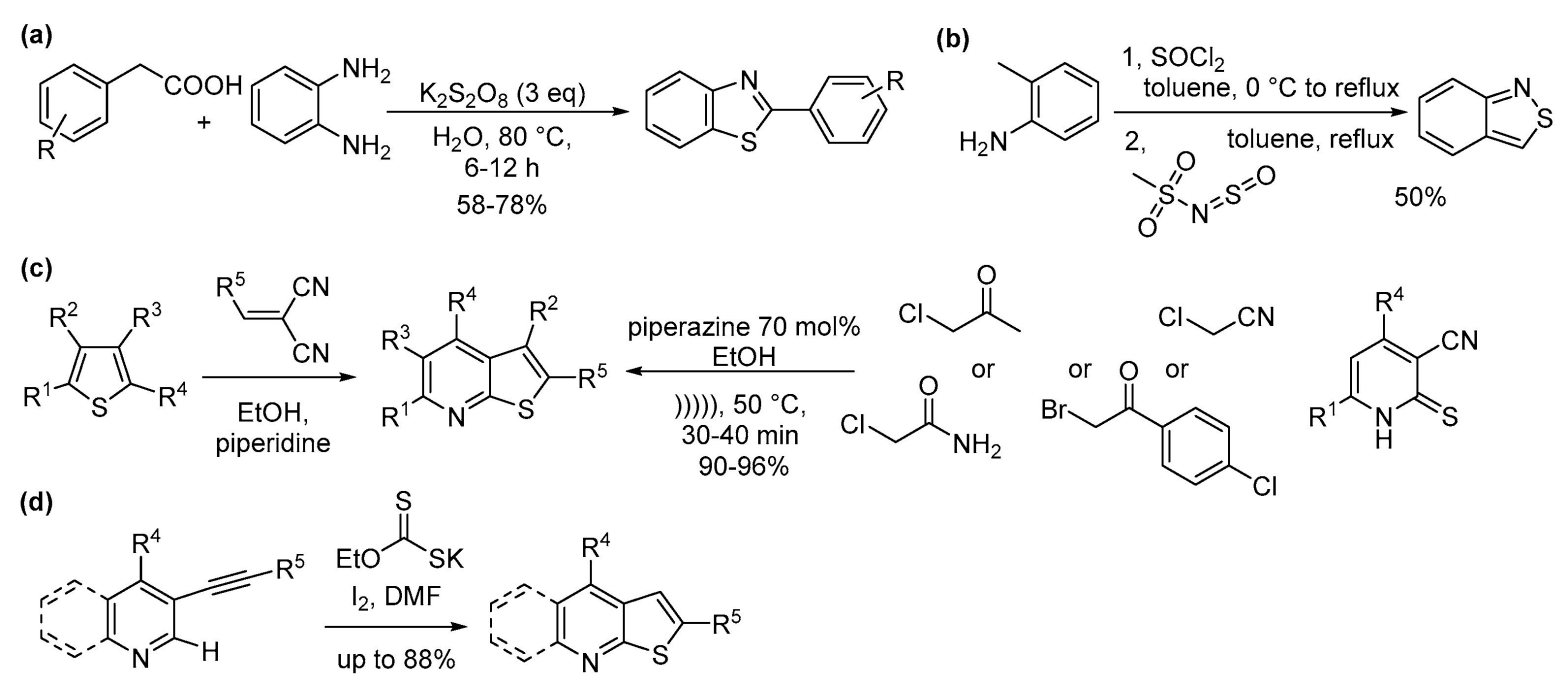
Environmentally friendly synthetic protocols for generating benzothiazole, benzoisothiazole and thieno[2,3-b]pyridine derivatives.

As part of the development of a green gram-scale sildenafil protocol, Laha’s group introduced a new synthetic strategy for constructing the 2-arylbenzo[*d*]thiazole fused ring system. (Scheme 21a) [167]. The innovation of the procedure is the use of arylacetic acid as an acyl source in the formation of the key pyrrazolo[4,3-d]pyrimidin-7-one ring. In addition, the organic solvent was replaced by an aqueous medium. In the presence of the oxidant K_2_S_2_O_8_, a number of additional 2-arylbenzo[d]thiazoles were synthesized using the same protocol in moderate to good yield (58–78%). An Agios Pharmaceuticals patent illustrated a new family of compounds with synthesis procedures that activate pyruvate kinase, making it a potential therapeutic agent for a disease related to PKR function and/or PKM2 function, such as certain cancers, diabetes or obesity (Scheme 21b) [168]. Although the described two-step process did not require the use of metal, toluene as a solvent does not fully comply with the principles of green chemistry. Nossier and Anwar have presented a practical synthesis protocol on how to build a thieno[2,3-b]pyridine scaffold (Scheme 21c) [169]. Thiophene and thiazole often exert a strong antiproliferative effect against various human cell lines, so the development of an efficient and benign synthesis of these compounds and their derivatives is very important. Another environmentally benign synthesis protocol for thieno[2,3-b]pyridine derivatives was reported by Mekky and coworkers (Scheme 21c) [170]. Pyridine-2(1H)-thiones were used as key intermediates to convert into thieno[2,3-b]pyridine derivatives. Excellent yields (90–96%) were achieved in a reaction of pyridine-2(1H)-thiones and α-halo compounds containing acidic C-H bond using eco-friendly piperazine as catalyst with ultrasonic activation. In addition to thienopyridines, this protocol was applied to produce nicotinonitrile derivatives, also with excellent results. Both groups of compounds showed a strong antibacterial effect. The last example of thienopyridine synthesis was presented by He et al. (Scheme 21d) [171]. In this metal-free highly selective cascade thiolation and cyclization reaction, EtOCS_2_K was used as a sulfur source in the presence of molecular iodine. Direct C-H functionalization of alkynylpyridine (and alkynylquinoline) derivatives resulted in the formation of thienopyridines (and thienoquinolines) in good yields of up to 88% and showed broad substrate tolerance.

**Scheme 22:**
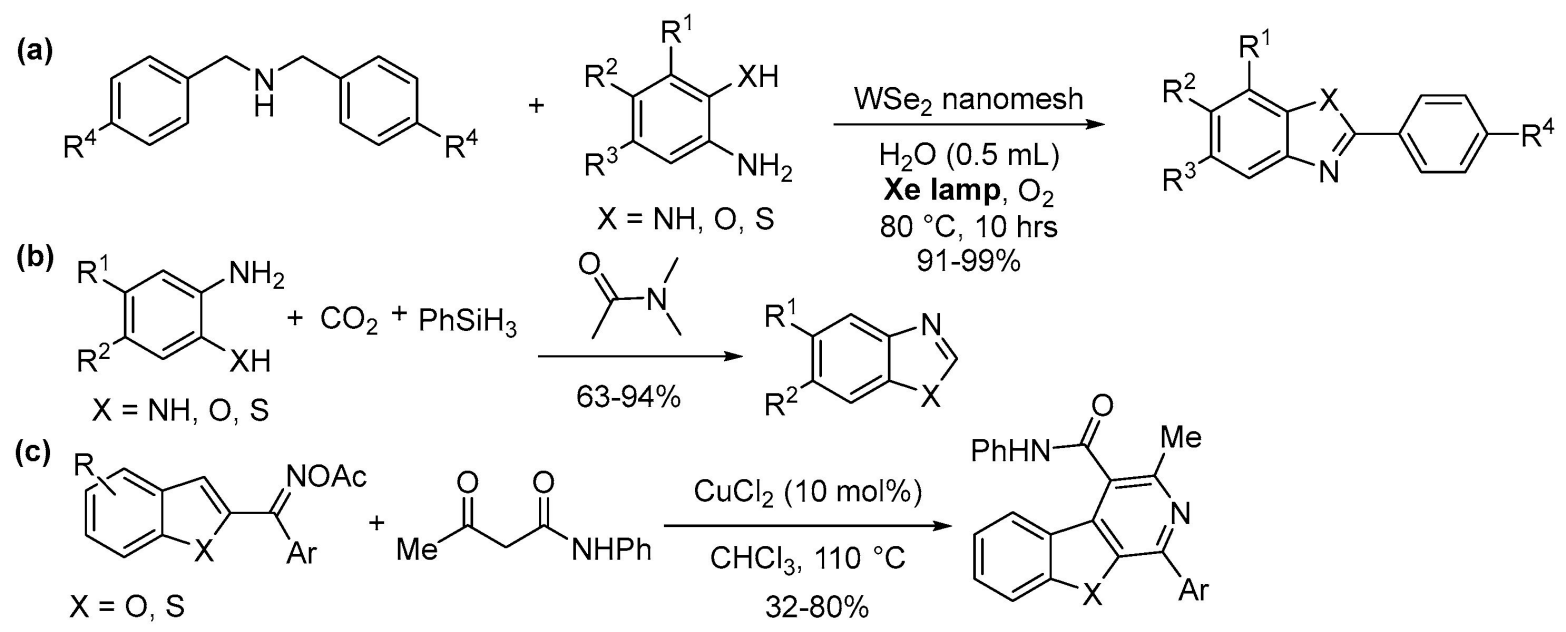
Environmentally benign synthesis protocols to generate benzimidazoles, benzoxazoles and benzothiazoles.

Finally, some protocols are presented that can be either used for the synthesis of fused 1*N*-heterocycles containing another nitrogen, oxygen or sulfur. For example, Chai et al. designed and synthesized a WSE_2_ nanomesh material that showed excellent photocatalytic activity in the oxidative coupling reaction of dibenzylamine and 2-amino/hydroxy/mercaptoaniline to produce benzimidazoles, benzoxazoles and benzothiazoles (Scheme 22a) [172]. The products were obtained in excellent yields (91–99%) under benign conditions, using only water as solvent and oxygen as an oxidizing agent, in the presence of visible light. The catalyst showed excellent stability over several catalytic cycles. A highly efficient catalyst-free green procedure was also developed to generate various N-containing benzoheterocycles in the presence of CO_2_ (Scheme 22b) [173]. The reaction conditions are decisive in terms of selectivity, such as the solvent, CO_2_ pressure, time, temperature and the hydrosilane quantity. Finally, Rong et al. presented a synthetic protocol for the annulation reaction of ketoxime acetates and acetoacetanilide in the presence of a copper catalyst (Scheme 22c) [174]. The simple, inexpensive synthetic strategy resulted in the desired benzofuro- and benzothieno[2.3-c]pyridines in good yields and with good substrate tolerance. Overall, all three processes are great examples of environmentally friendly methods to produce the therapeutically significant benzimidazole, benzoxazole and benzothiazole moiety.

## 5. Two-Nitrogen-Containing-Condensed Heterocycles with Multiple N Atoms

### 5.1. Benzimidazoles and Indazoles

Benzimidazole and its derivatives are explicitly acknowledged as important skeletons which possess various biological activities such as anticancer activity [175], antihypertensive [176], anti-inflammatory [177], antimicrobial [178], antioxidant activity [179] and anticoagulant activity [180]. Due to its significant role in drug applications, extensive efforts were devoted to the green synthesis of benzimidazoles.

**Scheme 23:**
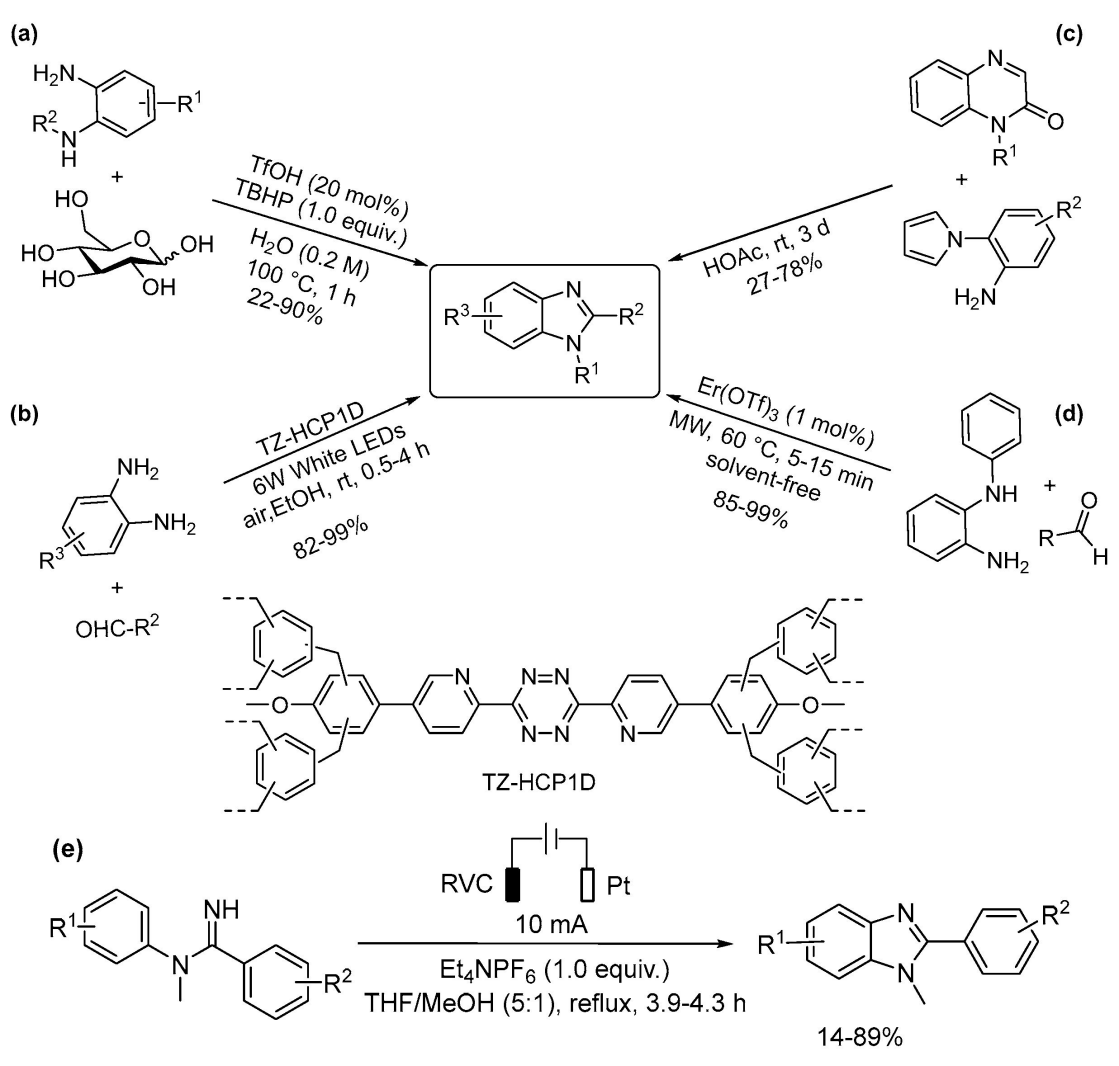
Green synthesis of benzimidazoles via non-traditional activation methods.

Raja et al. [181] developed an oxidative cyclization strategy by using the efficient C1 synthon _D_-glucose for the synthesis of benzimidazoles with yields 22–90% from *o*-phenylenediamines (Scheme 23a). This protocol possesses many advantages including short reaction time, broad functional group tolerance, metal-free, using environmentally benign solvent, water and bio-renewable methine sources. Hypercrosslinked polymers (HCPs) are of great interest due to their lightweight properties and high surface areas which makes them promising materials for catalysis [182]. An et al. successfully constructed a versatile photocatalyst TZ-HCP1D through Friedel–Crafts alkylation reaction (82–99% yield) (Scheme 23b) [183]. TZ-HCP1D was proved to be efficient in photocatalytic benzimidazole synthesis in EtOH. The heterogeneous system is considered green because of avoiding the use of metals, strong oxidants, acid and high temperature. In 2000, an unexpected rearrangement was discovered by Kalinin et al., when they mixed *o*-phenylenediamine with 3-benzoylquinoxalin-2(1*H*)one in boiling acetic acid [184] and obtained a benzimidazole derivative. Based on this finding, many valuable studies have been carried out. Among them, Li et al. explored a simple and environmentally friendly protocol to synthesize benzimidazoles [185]. A series of substituted products were obtained in moderate to good yields 27–78% with the only requirement of the solvent AcOH at room temperature (Scheme 23c). Since the clinically available benzimidazole-based drug is crucial, a rapid, cheap, clean and environmentally sustainable method for its synthesis has been reported. 1,2-disubstituted benzimidazoles (85–99% yield) were synthesized under microwave-assisted conditions without solvents, but 1% Er(OTf)_3_ was required as an efficient and environmentally mild catalyst (Scheme 23d) [186]. In recent years, dehydrogenative cross-coupling reactions under electrochemical oxidative conditions have gained significant attention [187,188]. An efficient and sustainable method that utilized electricity as a green reagent was developed for the synthesis of benzimidazoles (14–89% yield) through dehydrogenative cyclization of *N*-aryl amidines (Scheme 23e) [189]. The tandem cyclization was conducted without catalysts, oxidants or additives, generating H_2_ as the only byproduct. Another catalyst-free room temperature synthesis of isoquinoline-fused benzimidazoles was recently reported using 2-alkynylbenzalde- hydes and *o*-phenylenediamines in good to excellent yields in a green solvent, EtOH [190].

**Scheme 24:**
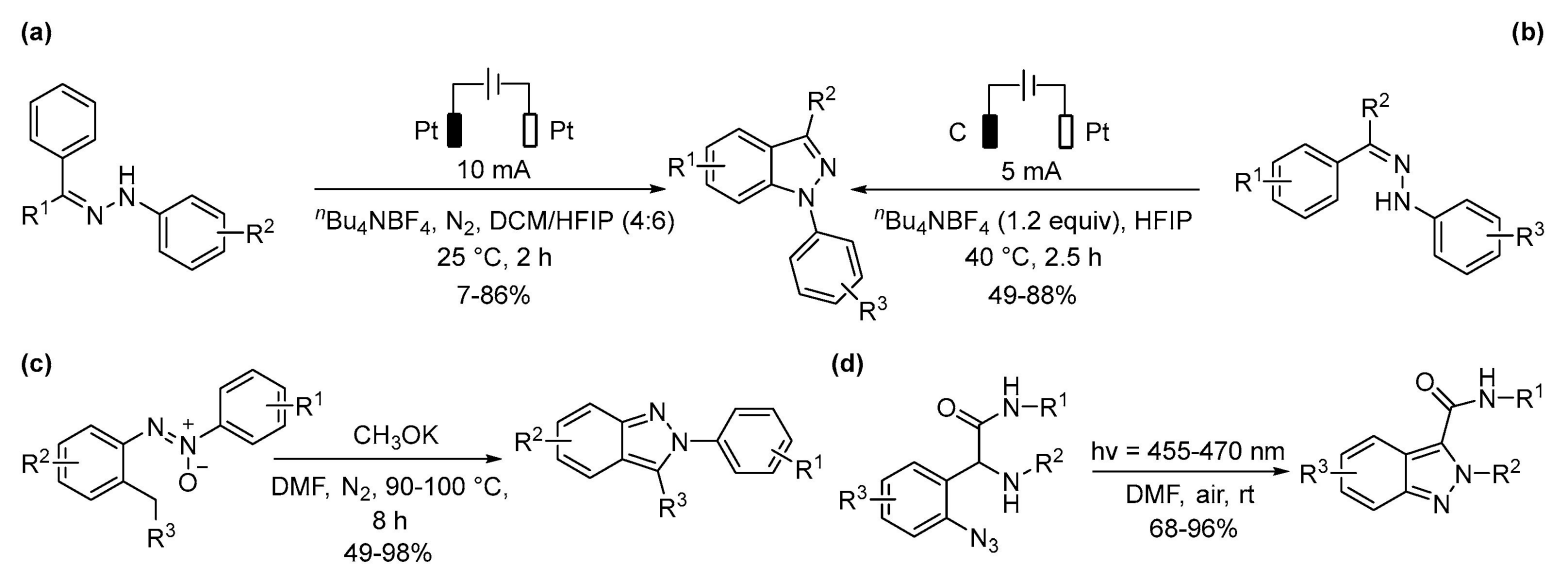
Facile and efficient synthetic methods to access 1*H*-indazole and 2*H*-indazole.

Substantial efforts have been made toward designing facile and efficient synthetic methods to access indazoles due to their biological and pharmaceutical applications [191]. 1*H*-indazole and its tautomer 2*H*-indazole have been known as crucial units in many pharmaceuticals [192,193]. A catalyst-free and chemical oxidant-free electrochemical synthesis of 1*H*-indazoles (7–86% yield) was demonstrated by Wan and co-workers (Scheme 24a) [194]. This indazole formation involves electrochemical oxidative radical Csp^2^–H/N–H cyclization of arylhydrazones. Cyclic voltammetry indicates that DCM/HFIP is essential in which the substrate has the lowest oxidation potential. Pt anode and *n*Bu_4_NBF_4_ electrolyte were the most effective for the activation of arylhydrazones.

Zhang et al. [195] reported another metal- and oxidant-free electrochemical protocol for the synthesis of 1H-indazoles from easily available hydrazones. This series of reactions was carried out in an undivided cell which was equipped with a carbon anode, a Pt cathode and *n*Bu_4_NBF_4_ electrolytes (49–88% yield) (Scheme 24b). It is worth noting that a wide range of functional groups are tolerated, and gram-scale reactions were accomplished under mild conditions. 2*H*-indazoles are important scaffolds in medicinal chemistry, exhibiting a broad range of pharmacological and biological activities such as antimicrobial and anti-inflammatory [196]. In particular, 2-aryl-2*H*-indazoles are crucial scaffolds found in various biologically active molecules. Additionally, its derivatives display attractive fluorophoric properties for cellular imaging [197]. Jin et al. synthesized several fluorescent and bioactive 2*H*-indazoles molecules via base-catalyzed benzylic C–H deprotonation and cyclization without using transition-metal catalysts (Scheme 24c) [198]. This synthetic strategy employs inexpensive CH_3_OK as the base and has good tolerance for halogen, electron-neutral methyl, electron-donating or electron-withdrawing groups, and provides the products in good to excellent yields, 49–98%. Liu et al. [199] reported a green and sustainable method to access 2H-indazole-3-carboxamides in moderate to excellent yields (68–96%) (Scheme 24d). Inspired by the Davis–Beirut reaction for the conversion of aromatic nitro compounds to *N*-aryl 2*H*-indazoles [200], a visible-light-driven N–N coupling of aryl azides to 2*H*-indazole-3-carboxamides has been developed without the presence of a photocatalyst and external additive addition. These reactions were carried out at room temperature under air, possessing additional advantages such as the broad substrate scope, excellent functional group compatibility and environment friendliness.

### 5.2. Carbolines

**Scheme 25:**
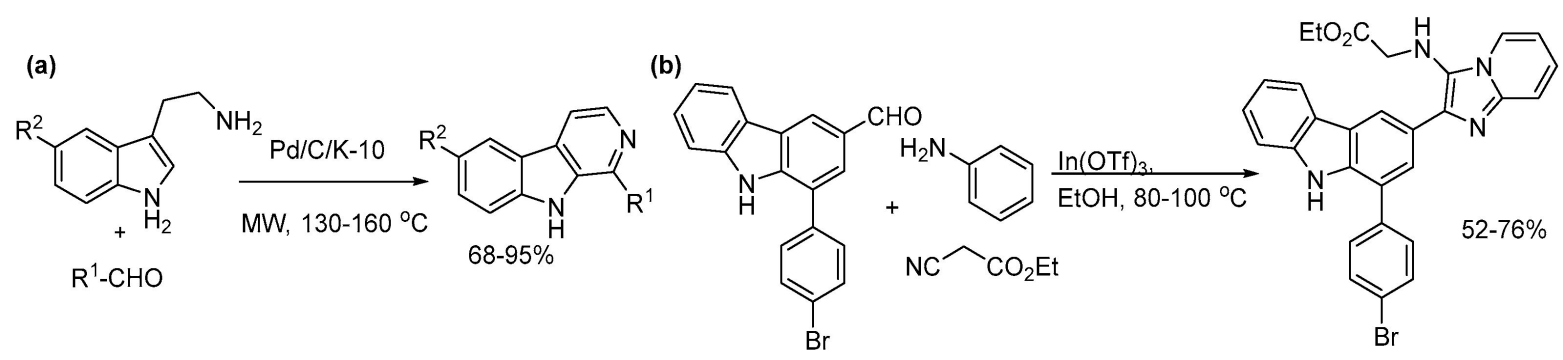
Catalytic syntheses of β–carbolines and derivatives.

Carbolines are three-ring heterocycles with two N-atoms one in the middle ring and another in either side rings, β-carbolines being the most common [201]. These compounds exhibit multiple types of biological activities, such as being multifunctional anti AD compounds [202], anticancer agents [203] or angiogenesis inhibitors [204]. A combined heterogeneous catalytic microwave-assisted synthesis was developed by using a Pd/C and K-10 montmorillonite-containing bifunctional catalyst. Tryptamines and carbonyl compounds were reacted to form β-carbolines in a green Pictet–Spengler cyclization in a three-step domino sequence affording the products in good to excellent yields (Scheme 25a) [205]. Another method used In(OTf)_3_ catalysis in a multicomponent reaction, using the Groebke–Blackburn–Bienayme (GBB) reaction [206]. The method provided good yields for the products and possessed several green advantages, such as operational simplicity, high atom economy, structural diversity, easy purification and the use of EtOH, a green solvent (Scheme 25b).

### 5.3. Quinazoline, Quinoxaline, Cinnoline

Quinazolines are important nitrogen-containing heterocycles many of them are bioactive natural and synthetic products of pharmaceutical interest with diverse properties such as antimicrobial [207], antiviral [208], antimalarial [209], antituberculosis [210], etc.

**Scheme 26:**
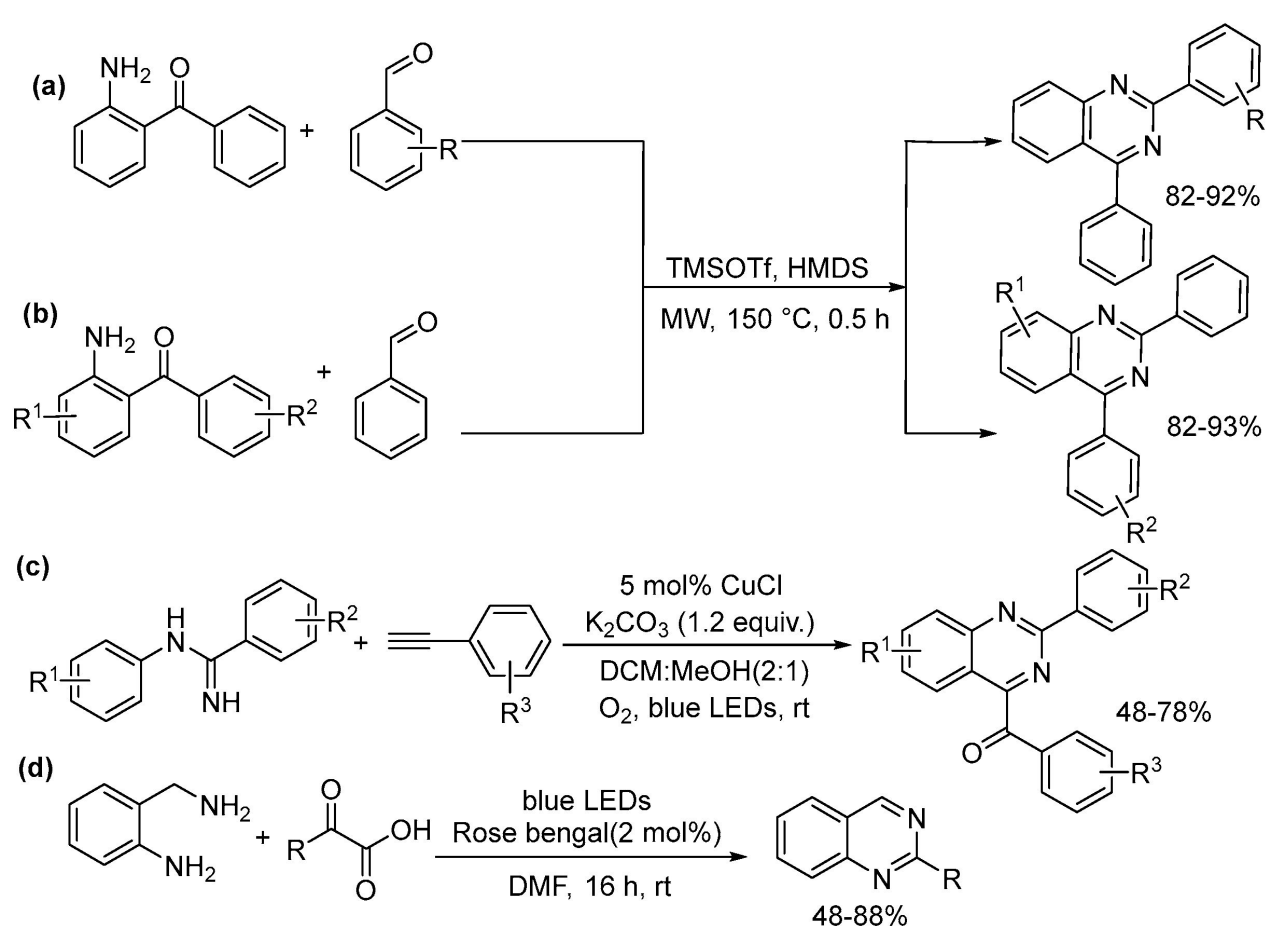
Green synthesis of functionalized quinazolines under microwave- (**a**,**b**) and visible light (**c**,**d**) activation.

Chan et al. [211] constructed substituted quinazolines from functionalized 2-amino-benzophenones and aromatic aldehydes (Scheme 26a,b). This efficient and mild protocol employed TMSOTf/hexamethyldisilazane (HMDS) catalytic system under neat, metal-free and microwave-assisted conditions. Notably, HMDS served as N sources and the reaction was catalyzed by TMSOTf, generating gaseous ammonia in situ. Most protocols for C–H functionalization/C–H annulation use transition metal catalysts under conventional thermal conditions. Owing to its common drawbacks, Charpe et al. [212] reported the visible light-initiated copper-catalyzed oxidative Csp^2^–H annulation of amidines with terminal alkynes to form 2,4-disubstituted quinazolines using molecular O_2_ as an oxidant at room temperature (48–78%; Scheme 26c). These reactions were carried out under mild conditions, which did not require any expensive, toxic external photocatalysts or organic oxidants. Water is the only byproduct of the reactions. In terms of green chemistry metrics, the E-factor is ~1.9 times better than that of the reported thermal method. Another transition metal catalyst- and additive-free quinazoline synthesis has been described by Yang et al. [213]. In this study, visible-light catalysis was applied as a green and clean way to access the coupling, decarboxylation and cyclization of 2-aminobenzylamine and *α*-ketoacids under room temperature in air (48–88% yield; Scheme 26d).

Quinoxalines and their derivatives have gained a great deal of attention in medicinal chemistry due to their diverse biological activities [214]. Additionally, quinoxaline derivatives have been reported for applications in fields such as organic semiconductors [215], dyes [216], organic photovoltaics [217] etc.

**Scheme 27:**
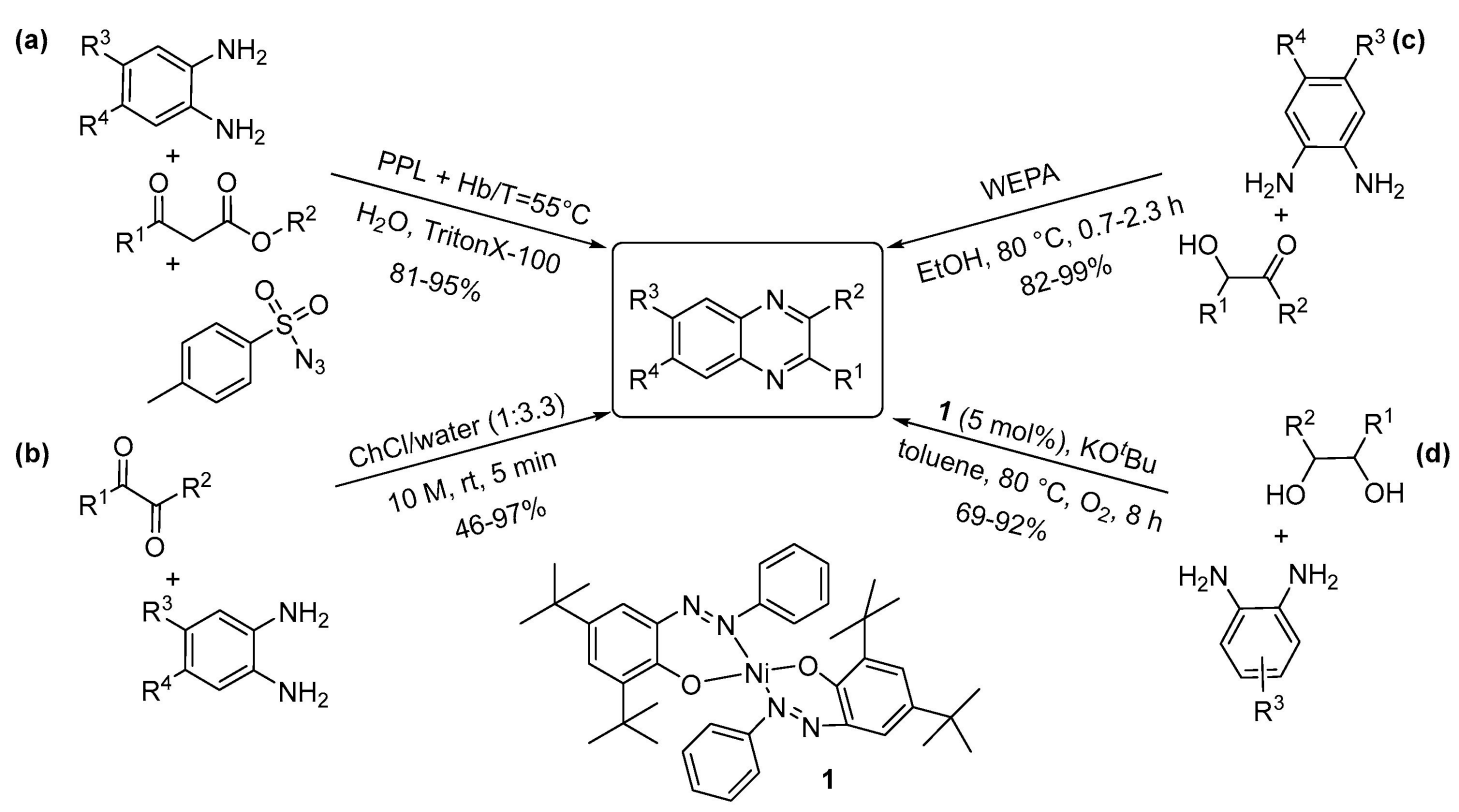
Environmentally benign and sustainable method for the synthesis of functionalized quinoxalines.

Li et al. [218] designed an efficient dual-protein (lipase and hemoglobin) system which successfully catalyzed the regioselective synthesis of quinoxalines in water. This system represents an environmentally benign, efficient and regioselective synthesis of quinoxalines. In this green and efficient protocol, lipase was used to access the in situ generations of diazodicarbonyls by a diazo transfer reaction to 1,3-dicarbonyl compounds with sulfonyl azides (Scheme 27a). The obtained *α*-diazo carbonyl compounds and 1,2-diamines were catalyzed by hemoglobin to afford the condensed quinoxaline products in excellent yield (81–95%). It is worth noting that this method can be easily scaled up, maintaining moderate regioselectivity.

Natural deep eutectic solvents (NADESs) are considered functional liquid media which can dissolve natural or synthetic chemicals of low water solubility. Owing to the special properties such as high biodegradability and biocompatibility, NADESs can serve as alternative candidates for organic solvents and ionic liquids [219]. Lupidi et al. [220] reported a sustainable and rapid method for the synthesis of functionalized quinoxalines, via the condensation of 1,2-dicarbonyls with 1,2-diamino compounds in a ChCl/water NADES in 5 min at room temperature (46–97%) (Scheme 27b). The involvement of NADESs enabled the fast activation of reactants, providing pure enough products to avoid further purification. Based on universal sustainable development goals, reusable biomass-based resources for organic synthesis are encouraged to develop cleaner, sustainable and safer chemistry [221]. The water extract of pomegranate peel ash (WEPA) was applied to catalyze a condensation–cyclization–oxidation process between *α*-hydroxy ketones and 1,2-diamines, affording quinoxalines with excellent yield (82–99%) at 80 °C in air (Scheme 27c). This sustainable protocol has many advantages, such as deploying oxygen from air, using recrystallization for purification in an aqueous medium and the ability to be scaled up. Bains et al. [222] investigated a bis(azophenolate) nickel complex in quinoxaline synthesis. The inexpensive, air-stable nickel catalyst successfully yielded quinoxalines (69–92%) under aerobic atmosphere at a mild reaction temperature of 80 °C via double dehydrogenative coupling of diamine and diol (Scheme 27d). A similar, but even more environmentally benign process provided quinoxalines in nearly quantitative yields in a catalyst and solvent-free process [223].

**Scheme 28:**
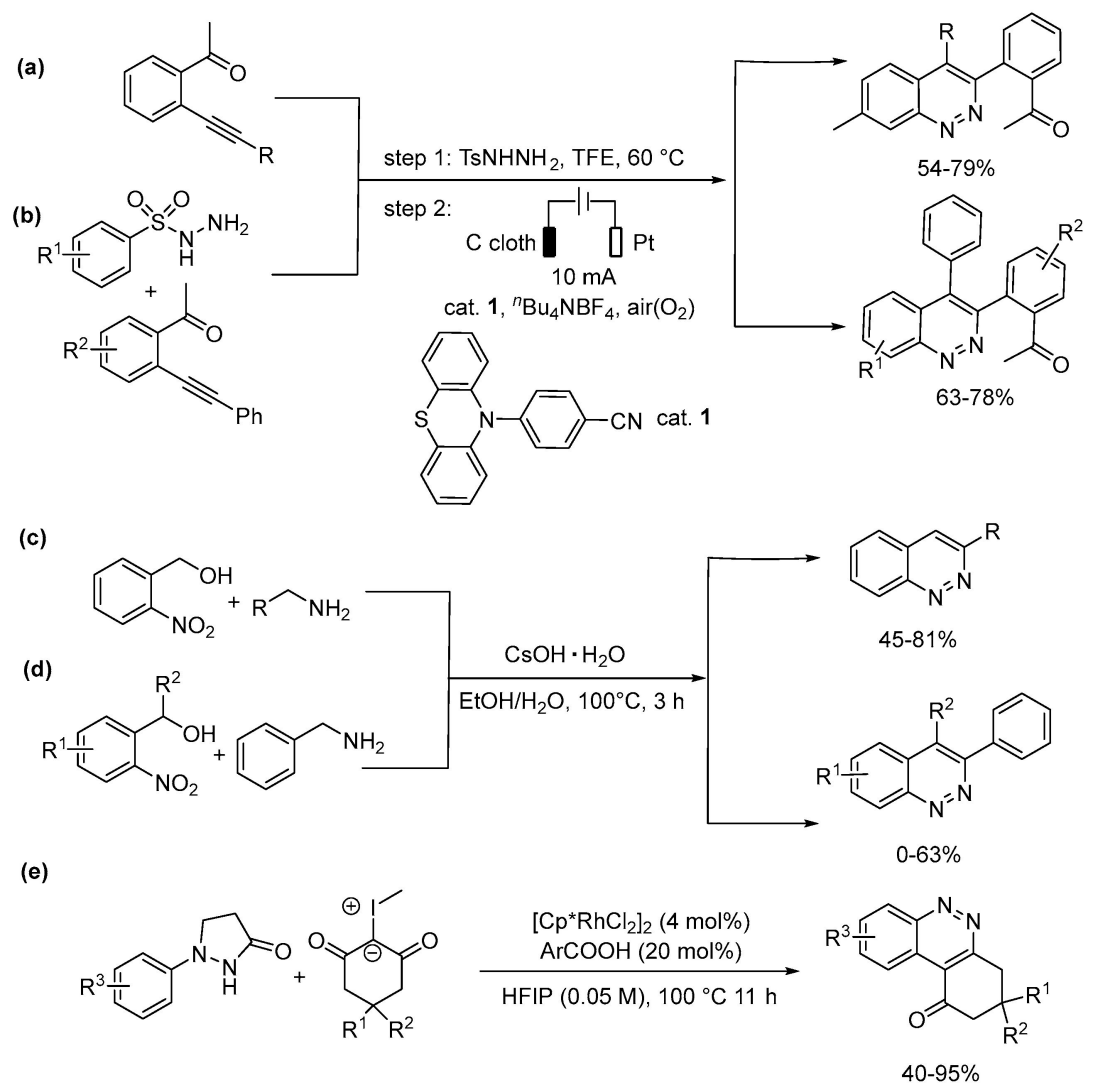
Straightforward access to cinnolines under mild reaction conditions.

Cinnolines are vital building blocks that are present in many compounds with considerable pharmaceutical properties such as antibacterial, antifungal, antimalarial, anti-inflammatory, analgesic, anxiolytic and antitumor activities [224], creating considerable interest in their preparation. Cai et al. [225] introduced a straightforward synthesis of cinnoline derivatives through aerobic electrochemical oxidation–cyclization and migration under metal-free conditions. This methodology utilized electrochemistry to promote a one-pot two-step cascade cyclization of *ortho*-alkynyl acetophenones and sulfonyl hydrazides (Scheme 28a,b). Moreover, this strategy has many benefits such as good to excellent yield 54–79%, broad functional group tolerance, good step economy and green reaction conditions.

A transition-metal-free intramolecular redox cyclization reaction [226] was developed for the straightforward synthesis of cinnolines from 2-nitrobenzyl alcohol and benzylamine by a simple treatment with C_S_OH·H_2_O in EtOH/H_2_O (45–81% yield; Scheme 28c). This method involves the formation of intermediate 2-nitrosobenzaldehyde and (*E*)-2-(2-benzylidenehydrazinyl) benzaldehyde which play an important role in the intramolecular redox cyclization. A rhodium-catalyzed [4+2] cyclization of readily available pyrazolidinones and iodonium ylides [227] was applied to synthesize cinnolines (Scheme 28d). The reactions were carried out in HFIP with 2,4,6-trimethylbenzoic acid additive. Moderate to excellent yields (40–95%) and broad scope were achieved under mild reaction conditions.

### 5.4. N and Other Heteroatoms

**Scheme 29:**
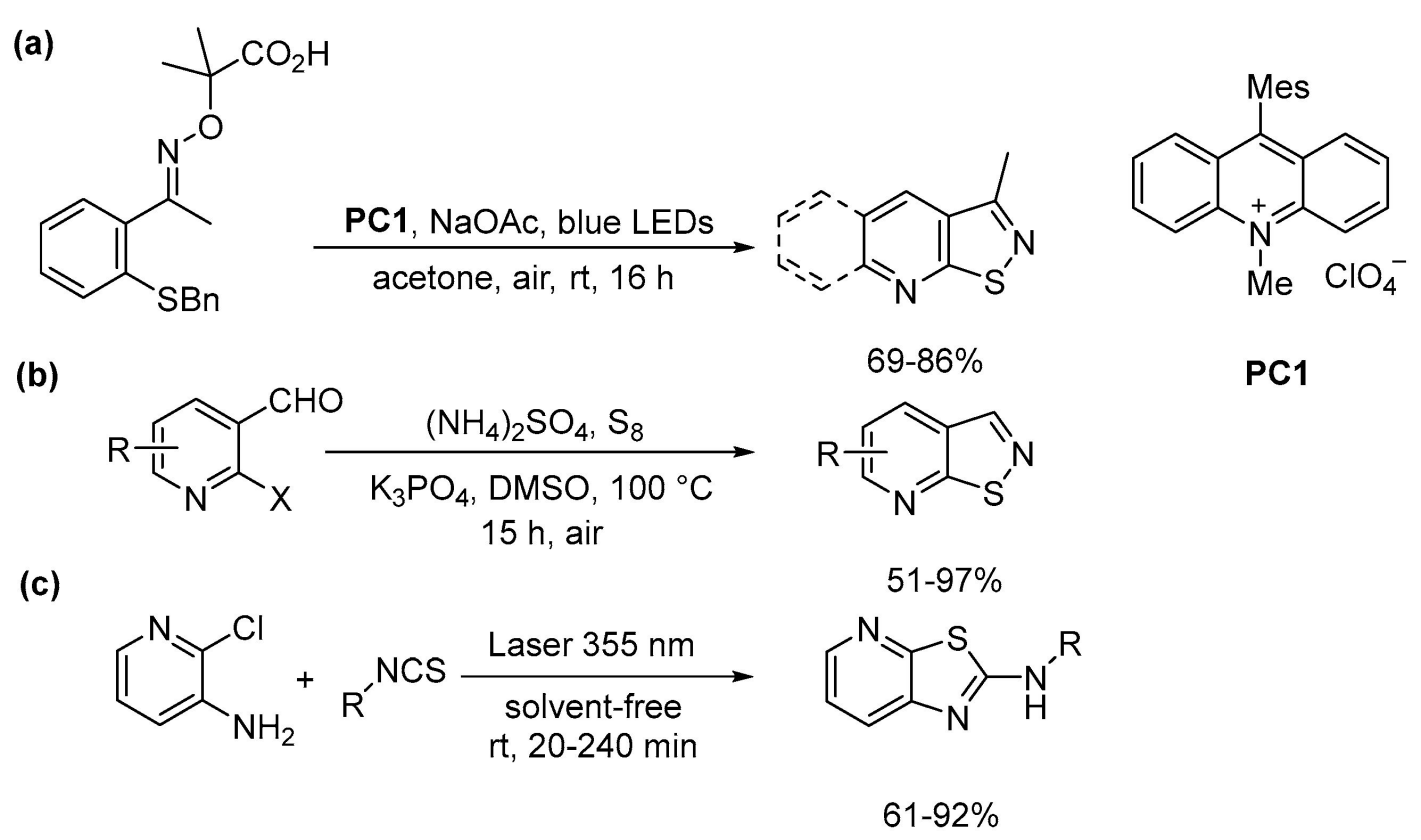
Sustainable synthesis of isothiazolo[5,4-b]pyridines (**a**,**b**) and phenylthiazolo[5,4-b]pyridines (**c**).

Heterocyclic compounds having isothiazole moiety exhibit analgesic, anorectic and antidepressant activities. Traditional methods for the synthesis of isothiazolo[5,4-b]pyridines, however, require high temperatures (250 °C) [228]. To address such issues, Cabrera-Afonso et al. [229] disclosed a sustainable synthesis of isothiazoles from α-imino-oxy acids. This efficient method applied an organo-photoredox generation of iminyl radicals by oxidative single-electron transfer (SET) to the formation of N–S bonds (up to 86%). The reactions were carried out under air and 450 nm single LED at room temperature without transition metals or stoichiometric oxidants (Scheme 29a). Wang et al. [230] used elemental sulfur and ammonium sulfate as heteroatom components to synthesize isothiazolo[5,4-b]pyridines via a three-component reaction under transition metal-free conditions (Scheme 29b). Elemental sulfur has the advantages of being inexpensive, industrially available, stable, non-volatile, easily stored, non-hygroscopic and non-toxic in nature compared to other sulfur sources such as KSCN, thiadiazole, etc. These reactions are moisture and oxygen tolerant and can easily be scaled up, providing products in good to excellent yields 51–97%. Jemili et al. [231] described a new laser-assisted synthesis of phenylthiazolo[5,4-b]pyridine derivatives (Scheme 29c). This rapid and straightforward approach benefits from the absence of solvent, yielding 61–92% product. It was also noted that increasing laser energy input increased the yields. Moreover, the conditions are solvent-, metal- and base-free.

## 6. Three- and Four-N-containing Condensed Heterocycles

### Purine, 3H-imidazo[4,5-b]pyridine, Imidazo[1,2-c]pyrimidine

**Scheme 30:**
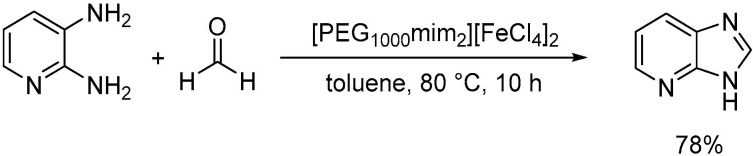
Synthesis of 3H-imidazo[4,5-b]pyridine from aryldiamines with formaldehyde.

Polyethylene glycol-based dicationic ionic liquid [232] has been applied in a green protocol to synthesize 3H-imidazo[4,5-b]pyridine. Fe(III)-based PEG_1000_ dicationic imidazolium ionic liquid ([PEG_1000_mim_2_][FeCl_4_]_2_)/toluene biphasic system served as a medium and catalyst as well to promote the reaction effectively providing 78% yield (Scheme 30) [233]. As a further green advantage the solvent could be recycled and maintained good catalytic activity after seven runs.

**Scheme 31:**
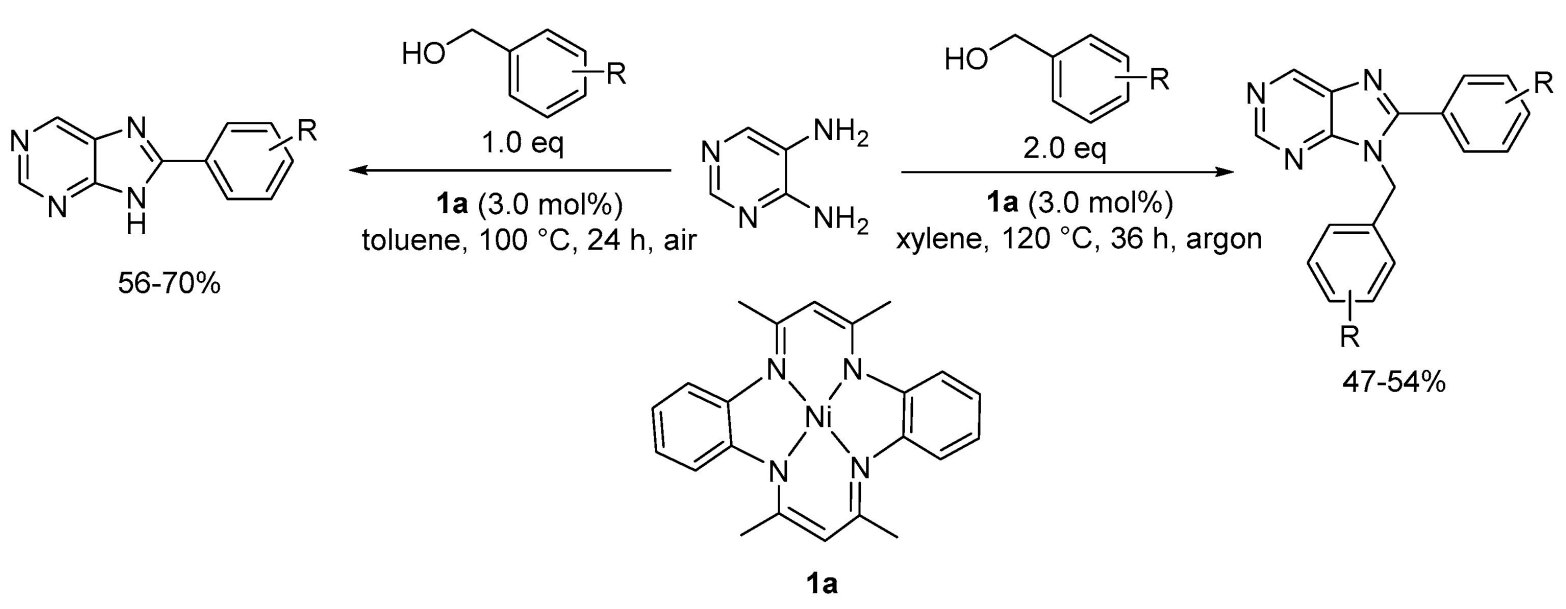
Synthesis of various purines via acceptorless dehydrogenative coupling.

Chakraborty et al. reported a sustainable nickel-catalyzed methodology for the synthesis of various purines via dehydrogenative functionalization with benzyl alcohols [234]. The bench stable catalyst was easily prepared and could catalyze the selective formation of 8-substituted (56–70% yield) and 8,9-disubstituted (47–54% yield) purine derivatives under different reaction conditions. Moreover, 8-substituted purine derivatives were obtained via using toluene as a solvent at 100 °C under air, while 8,9-disubstituted purine derivatives were obtained with xylene as a solvent at 120 °C under argon atmosphere (Scheme 31).

**Scheme 32:**
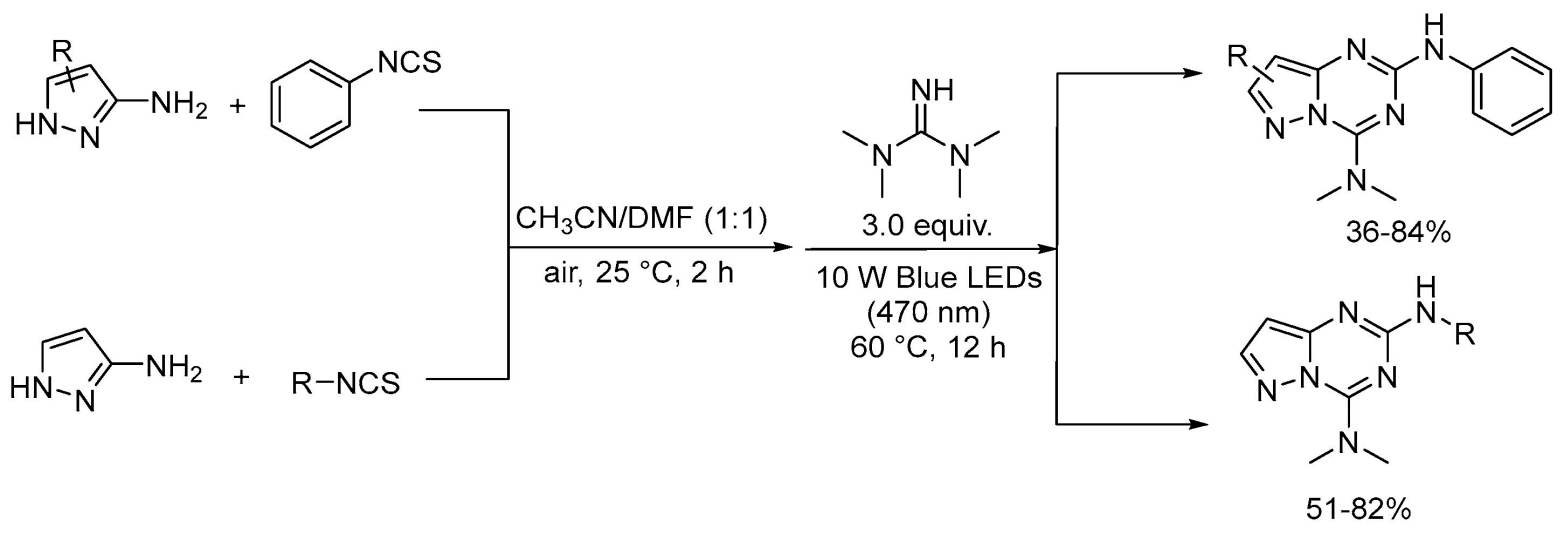
Visible-light-enhanced annulation approach to pyrazolo[1,5-a][1,3,5]triazine-2,4-diamines.

Guo et al. [235] reported an efficient synthesis of pyrazolo[1,5-*a*][1,3,5]triazine-2,4-diamines via a visible-light-enhanced annulation of 1*H*-pyrazol-3-amines and isothiocyanates [236]. This strategy generated in situ pyrazolthiourea intermediates in the first step, which then underwent a formal [4+2] annulation with 1,1,3,3-tetramethylguanidines(TMG) to give the corresponding products in 36–84% yield (Scheme 32). This method featured green advantages such as the use of a photocatalyst-, metal-, oxidant-, ligand-, base-free and broad substrate scope.

**Scheme 33:**
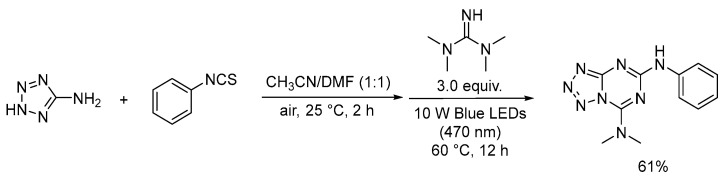
Synthesis of a potential biologically active compound under visible light activation.

Additionally, this protocol can be applied to synthesize a potential biologically active product (61% yield) when using 2*H*-tetrazol-5-amine as the substrate (Scheme 33).

## 7. Summary and Conclusions

As described above, the preparation of N-heterocycles is still at the forefront of organic synthesis research, although the focus has shifted toward the application of contemporary synthetic methods that comply with the major principles of green chemistry and engineering. In this work the highlight was placed on the use of catalytic methods and energy efficient activations by nontraditional methods, such as microwaves, ultrasounds, photo- and electrochemistry and high hydrostatic pressure. The protocols listed in this account are representative examples of the new developments illustrating the synthesis of the target compounds through a green and sustainable perspective.

## Data Availability

No new data were created.
